# Short-term effects of air pollutants on outpatients with psoriasis in a Chinese city with a subtropical monsoon climate

**DOI:** 10.3389/fpubh.2022.1071263

**Published:** 2022-12-22

**Authors:** Ting Wang, Yuanrui Xia, Xinhong Zhang, Nini Qiao, Susu Ke, Quan Fang, Dongqing Ye, Yinguang Fan

**Affiliations:** ^1^Department of Epidemiology and Biostatistics, School of Public Health, Anhui Medical University, Hefei, China; ^2^Department of Health Education, Anhui Provincial Center for Disease Control and Prevention, Hefei, China; ^3^Inflammation and Immune Mediated Diseases Laboratory of Anhui Province, Hefei, China

**Keywords:** meteorological factors, air pollutants, outpatients with psoriasis, distribution lag non-linear model (DLNM), time series analysis

## Abstract

**Introduction:**

Psoriasis is a common skin disease that seriously affects patients' quality of life. The association of air pollutants with psoriasis, and the extent of their effects remains unclear.

**Methods:**

Based on a distributed lag non-linear model, this study explored the short-term effects of air pollutants on outpatients with psoriasis in Hefei, China, between 2015 and 2019 by analyzing the exposure–lag–response relationship, after controlling for confounding influences such as meteorological factors, long-term trends, day of the week, and holidays. Stratified analyses were performed for patients of different ages and genders.

**Results:**

The maximum relative risks of psoriasis outpatients' exposure to SO_2_, NO_2_, and O_3_ were 1.023 (95% confidence intervals (CI): 1.004–1.043), 1.170 (95% CI: 1.046–1.307), and 1.059 (95% CI: 1.030–1.090), respectively. An increase of 10 μ*g*/*m*^3^ of NO_2_ was associated with a 2.1% (95% CI: 0.7–3.5%) increase in outpatients with psoriasis, and a decrease of 10 μ*g*/*m*^3^ of O_3_ was associated with an 0.8% (95% CI: 0.4–1.2%) increase in outpatients with psoriasis. Stratified analyses showed that male subjects were more sensitive to a change in meteorological factors, while female subjects and outpatients with psoriasis aged 0–17 years old were more sensitive to a change in air pollutants.

**Discussion:**

Short-term air pollutant exposures were associated with outpatients having psoriasis, suggesting that patients and high-risk people with psoriasis should reduce their time spent outside and improve their skin protection gear when air quality is poor.

## Introduction

Psoriasis is an immune-mediated chronic papulosquamous disease that affects the skin and/or joints, creating a considerable burden for individuals and society. It presents many challenges including chronicity, disfiguration, disability, and associated comorbidities (1). Psoriasis has been reported worldwide, and its prevalence varies from 0.14 (East Asia) to 1.99% (Australasia) (2). In 2010, a cross-section study found that the overall standardized prevalence of psoriasis was 0.47% in six cities in different provinces in China (3). As a systematic analysis estimated, although the incidence of psoriasis was inconspicuous among counties in China, the number of people that suffered from psoriasis was as high as 2.3 million, the third highest in the world (2). A skin disease burden analysis revealed that psoriasis plays a non-negligible role in the overall disease burden among people over 50 years of age in China, suggesting that psoriasis is a notable disease among middle-aged and elderly people that necessitates intervention (4). Psoriasis cannot be cured currently; as a chronic inflammatory disease, psoriasis has a long course and is prone to recurrence in most cases, making the patients visit a doctor many times.

The pathogenesis and inducement of psoriasis are complicated. In addition, internal factors, such as heredity, immunity, and metabolism, and environmental factors have been thought to play a role in psoriasis. Climate change and exposure to sunshine have been considered to affect the prevalence of psoriasis, as cutaneous psoriasis and psoriatic arthritis tend to worsen in the winter and improve in the summer under different amounts of sunlight (5). In 1984, a large-scale investigation of psoriasis was carried out in China, and it was found that the prevalence of psoriasis in northern China was higher than that in southern China, with 35° N latitude as the dividing line (6). A systematic review has found that the prevalence of psoriasis increases as the distance from the equator increases (7), providing evidence for a latitudinal correlation in psoriasis.

Several studies showed that psoriasis exists seasonally, with a higher risk of onset in the spring and autumn (8, 9). Moreover, the colder the weather, the easier psoriasis symptoms exacerbate (10). The worsening symptoms of psoriasis in a considerable percentage of patients have been attributed to the low humidity, dry environment, and low temperatures in the spring and autumn (5). The use of ultraviolet light for treating psoriasis, and the function of photochemotherapy in restoring dysfunctional Th17 cells/regulatory T cells, reveal a positive effect of sunlight on the disease (11). A study found that the concentrations of pollutants such as CO, NO_2_, other nitrogen oxides, benzene, PM_10_, and PM_2.5_ were significantly higher in the 60 days before a psoriasis flare, according to a questionnaire survey (12). Accordingly, the daily number of outpatients with psoriasis appears to change depending on meteorological factors and concentrations of air pollutants.

In recent years, many studies have attempted to determine the mechanisms of air pollutants acting on skin diseases. An experimental study found that carbon particle treatments upregulated psoriasis-related genes (13) and that pollutants can affect skin diseases by weakening the skin barrier or modifying skin absorption (14, 15). Several psoriasis-related skin bacteria are affected by NO_2_ (16, 17). O_3_ can activate cutaneous inflammasomes, which may induce inflammatory skin conditions (18). Furthermore, smoking may be a risk factor for psoriasis because benzo[a]pyrene could enhance scratch-induced CCL20 secretion (19). Polycyclic aromatic hydrocarbons can also influence skin diseases by affecting the selective growth of skin microbiome (20).

Understanding the role of environmental factors on psoriasis will help to manage this complex disease and reduce the disease burden on the health system. Time series analysis tends to be a good method to explore the association between environmental factors and worsening psoriasis symptoms. Since the effects of environmental factors on disease often act non-linearly, cumulatively, and hysteretically, it is suitable to use a distribution lag non-linear model (DLNM) for the analysis (21). DLNM has been used to explore the effects of environmental factors on diseases since 2010; many studies have applied the method to dermatoses, but few have applied it to the study of psoriasis. This study established a time series, based on the data of outpatients with psoriasis in two representative hospitals in Hefei from 2015 to 2019, and aimed to explore the influence of air pollutants *via* modification of meteorological factors.

## Materials and methods

### Study area

Hefei (31.87° N, 117.28° E) has an area of 11,445.1 km^2^ and is the capital of Anhui Province. It is a metropolis that has a substantial number of permanent residents (9,369,881 population, 15.35% of Anhui in 2020). Hefei is geographically part of the semitropics, and the spring season there begins in March. Hefei has an average annual temperature of 15.7°C, nearly 1,000 mm of annual rainfall, and more than 2,100 h of annual sunshine.

### Data source

Data on outpatients with psoriasis were collected from the First Affiliated Hospital of the University of Science and Technology of China and the First Affiliated Hospital of Anhui Medical University from 1 January 2015 to 31 December 2019, covering patients' demographic information, such as sex and age. The inclusion criteria for patients with psoriasis were in line with the Guidelines for the Diagnosis of Psoriasis in China, and the included patients belonged to the permanent population of Hefei. Exclusion criteria were patients who attended both hospitals at the same time or those who attended two hospitals two times within a short period. Using a stratified analysis, outpatients with psoriasis were divided into four groups according to age: 0–17 years old, 18–39 years old, 40–64 years old, and ≥ 65 years old (22, 23). The study was approved by the School of Public Health, Anhui Medical University Research Ethics Committee (No. 20200594).

Daily meteorological data were obtained from fixed monitoring stations set up by the Hefei Meteorological Bureau (http://data.cma.cn/), including the daily average relative barometric pressure (BP), wind speed (WS), average daily temperature (ADT), diurnal temperature range (DTR), relative humidity (RH), precipitation (PRE), and sunshine hours (SSH). The Hefei Environmental Protection Bureau (http://sthjj.hefei.gov.cn/) provided data on air pollutants, including sulfur dioxide (SO_2_), nitrogen dioxide (NO_2_), carbon monoxide (CO), ozone (O_3_), fine particulate matter (PM_2.5_), inhalable particulates (PM_10_), and air quality index (AQI).

### Statistical analyses

Spearman's rank correlation analysis was used to analyze the correlation among environmental factors, and strong correlations (*rs* > 0.8) were not incorporated into the model to avoid strong multicollinearity between variables. In this study, outpatients with psoriasis could be regarded as low-probability events, following a Poisson distribution; a Quasi–Poisson distribution was finally adopted as the function family because of the overdispersion of data (24). A generalized linear regression model (GLM) combined with the DLNM was used to study the non-linear, cumulative, and hysteretic effects of air pollutants on outpatients with psoriasis. The natural cubic splines (*ns*) function was used to control for meteorological factors, long-term trends (time), day-of-the-week (DOW) effects, holiday effects, and other confounding factors in the model. The degree of freedom (*df*) combination that minimizes the Akaike information criterion (AIC) was used as a parameter in the final model (25). The model that incorporates covariates was built as follows:


$$\begin{array}{l} E(Y_{t})=\alpha +cb\left(M,df,\text{lag},df\right)+\text{as}.\text{factors}\left(DOW\right)+\;\\ \text{ as}.\text{factors}(\text{holiday})+ns\left(\text{time},df^{*}year\right)+ns\left(X_{i},df\;\right) \end{array}$$

where *Y*_t_ refers to the observed number of outpatients with psoriasis, while E(*Y*_t_) represents the expected number of outpatients with psoriasis. α is an intercept, and *df* refers to the degree of freedom parameter. *M* represents the research variable; its exposure and lag dimensions are transformed by the cross basis (*cb*) function. To control the impact of short-term fluctuations on outpatients with psoriasis, DOW and holidays were considered as factors in this study, and the *ns* function was used to control long-term trends. In addition to the research variable *M*, meteorological factors and other air pollutants (*X*_*i*_) were incorporated into the model using the *ns* function to construct the multivariate models.

R 4.0.5 software was used to conduct all statistical analyses and create figures; the “dlnm” and “splines” packages were adopted in the model analyses. Contour and three-dimensional graphs were adopted to describe the effects of the factors on outpatients with psoriasis. The effects, expressed as the relative risk (RR) values, of variables on outpatients with psoriasis relative to the median on different lag days were observed, as well as the changing rules on lag days; a *p*-value of <0.05 was considered statistically significant.

## Results

### Characteristics of the data

**Figure 1:**
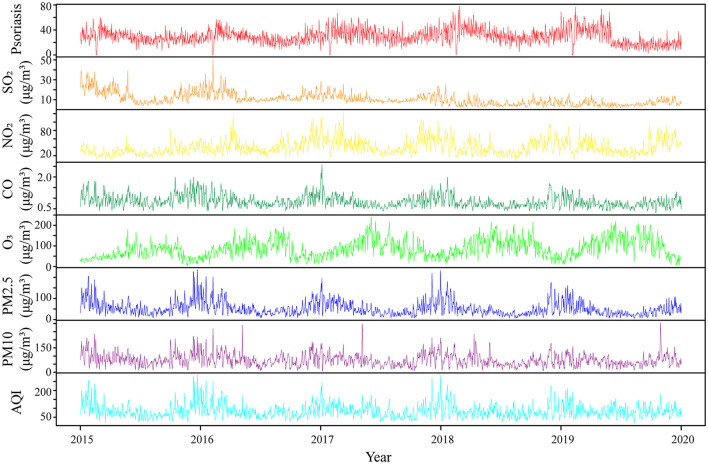
Time series characteristics of air pollutants and outpatients with psoriasis in Hefei city from 2015 to 2019. SO_2_, sulfur dioxide; NO_2_, nitrogen dioxide; CO, carbon monoxide; O_3_, ozone; PM_2.5_, fine particulate matter; PM_10_, inhalable particulates; AQI, air quality index.

A total of 54,064 visits were registered as outpatients with psoriasis in the two Hefei hospitals from 2015 to 2019, including 30,899 male subjects and 23,165 female subjects (with a ratio of 1.33:1), and an average daily visit number of 29.61. Outpatients with psoriasis aged 18–39 years (12, 26) accounted for the highest proportion (48.11%). The number of outpatients with psoriasis showed a periodic fluctuation at the annual level, which manifested as a gradual increase in the winter, reaching a peak in March, followed by a decrease. Meteorological factors and air pollutant concentrations showed regular periodicity at the annual level, and the statistical information and time series distribution are shown in Table 1, Figure 1, and Supplementary Figures S1, S2.

**Table 1:** Characteristics of outpatients with psoriasis, meteorological factors, and air pollutants in Hefei city from 2015 to 2019.

**Variables**	**Mean**	**SD**	**Minimum**	**P_25_**	**P_50_**	**P_75_**	**Maximum**
Outpatients with psoriasis	29.61	11.78	0.00	22.00	28.00	37.00	78.00
**Sex**							
Male	16.92	7.23	0.00	12.00	16.00	21.00	51.00
Female	12.69	5.76	0.00	9.00	12.00	16.00	35.00
**Age (years old)**							
0– <18	2.83	2.35	0.00	1.00	2.00	4.00	14.00
18– <40	14.25	6.21	0.00	10.00	14.00	18.00	40.00
40– <65	10.55	5.25	0.00	7.00	10.00	13.00	40.00
≥65	1.98	1.74	0.00	1.00	2.00	3.00	11.00
**Meteorological factors**							
BP (hPa)	1012.67	9.52	989.50	1004.50	1012.70	1020.00	1040.90
WS (m/s)	2.11	0.73	0.80	1.60	2.00	2.50	5.60
ADT (°C)	16.85	9.21	−6.20	8.60	17.60	24.50	34.80
DTR (°C)	8.39	3.77	0.80	5.50	8.30	11.10	19.80
RH (%)	76.51	11.99	39.20	68.70	77.00	85.70	98.30
PRE (mm)	3.18	8.88	0.00	0.00	0.00	1.60	134.00
SSH (h)	4.92	4.02	0.00	0.40	5.10	8.50	12.90
**Air pollutants**							
SO_2_ (μ*g*/*m*^3^) (24 h)	10.55	6.08	2.00	6.00	9.00	13.00	51.00
NO_2_ (μ*g*/*m*^3^) (24 h)	40.31	17.76	9.00	27.00	36.00	51.00	125.00
CO (μ*g*/*m*^3^) (24 h)	0.84	0.28	0.30	0.60	0.80	1.00	2.60
O_3_ (μ*g*/*m*^3^) (8 h)	86.32	44.01	4.00	52.00	79.00	115.00	241.00
PM_2.5_ (μ*g*/*m*^3^) (24 h)	52.29	33.41	6.00	29.00	44.00	66.00	237.00
PM_10_ (μ*g*/*m*^3^) (24 h)	76.17	38.90	8.00	48.00	71.00	97.00	308.00
AQI	85.61	38.68	14.00	59.00	78.00	104.00	287.00

### Correlation analysis

Supplementary Figure S3 shows Spearman's correlation analysis results for outpatients with psoriasis, meteorological factors, and air pollutants in Hefei city during 2015–2019. From 2015 to 2019, there was no correlation between outpatients with psoriasis and WS, DTR, PRE, and SSH (all with a *p*-value > 0.05). The number of patients was positively correlated with PM_10_, PM_2.5_, SO_2_, NO_2_, CO, and BP (all with *p* < 0.05), but negatively correlated with O_3_, ADT, and RH (all with *p* < 0.05). Correlation analysis of different meteorological factors and air pollutants found that there were strong collinearities between BP and ADT, CO and PM_2.5_, PM_2.5_ and PM_10_, PM_10_ and AQI (*r* > 0.8), which were not suitable for simultaneous inclusion in the model analysis.

### Association between air pollutants and outpatients with psoriasis

**Figure 2:**
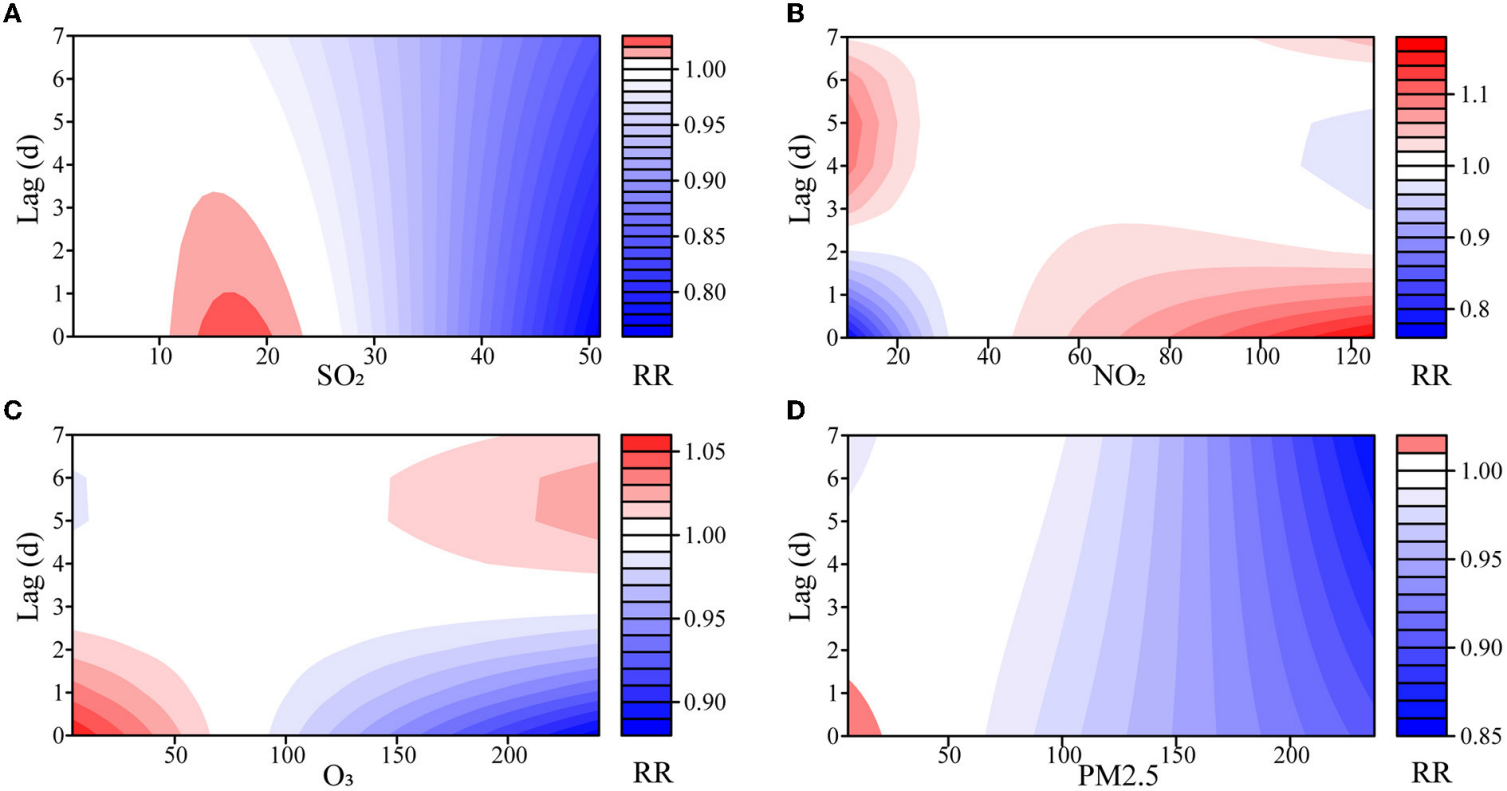
Exposure–lag–response association of meteorological factors and air pollutants on outpatients with psoriasis. **(A)** SO_2_, sulfur dioxide; **(B)** NO_2_, nitrogen dioxide; **(C)** O_3_, ozone; **(D)** PM_2.5_, fine particulate matter.

The effects of SO_2_ and PM_2.5_ on the number of outpatients with psoriasis were not statistically significant at low concentrations, but the RRs decreased with the lag days at high concentrations (Figure 2, Supplementary Figures S4–S7). Notably, the risk from SO_2_ increased modestly at 15 μg/m^3^ (RR = 1.023, 95% CI: 1.004–1.043). Although these effects lost their statistical significance on the fifth day, their cumulative effect did not (Supplementary Figure S7).

Both NO_2_ and O_3_ had opposite effects on the number of outpatients with psoriasis at low or high concentrations (Supplementary Table S1, Supplementary Figures S6, S7). At low concentrations, NO_2_ showed a dangerous effect on the fourth day, and the risk reached its maximum on the 6th day (RR = 1.099, 95% CI: 1.066–1.133). At high concentrations, NO_2_ showed a maximum risk effect on the 1st day (RR = 1.170, 95% CI: 1.046–1.307), and the effect decreased as the days lagged. O_3_ had the opposite impact, with a maximum effect of RR = 1.059 (95% CI: 1.030–1.090) at high concentrations. Similarly, the statistical significance of the effects of NO_2_ and O_3_ on outpatients with psoriasis gradually disappeared in the lag dimension, but almost all the cumulative effects were statistically significant (Supplementary Figure S7).

### Effect of change in air pollutant concentration

As shown in Supplementary Table S2 and Supplementary Figure S8, the median of each variable was defined as the reference point, and the concentrations of the air pollutants were then changed by 10 or 20 μ*g*/*m*^3^. Only NO_2_ manifested as a risk factor from either its single day or cumulative effects when increased by 10 μ*g*/*m*^3^ with RR = 1.021 (95% CI: 1.007–1.035), while other pollutants were mostly not risky or ineffective with respect to outpatients with psoriasis. When reduced by 10 μ*g*/*m*^3^, only O_3_ showed a dangerous effect on the 1st day with RR = 1.008 (95% CI: 1.004–1.012). NO_2_ was not risky on the 1st day and dangerous on the fourth day, and most of the other pollutants were not risky or ineffective for outpatients with psoriasis. Notably, the effects of SO_2_ appeared days after it was first increased by 20 μ*g*/*m*^3^.

### Stratified analyses

The stratified analysis found that the number of female outpatients with psoriasis was sensitive to increased SO_2_ concentrations, while male outpatients were sensitive to decreased SO_2_ concentrations. The decrease in the PM_2.5_ concentration negatively impacted female subjects (RR = 1.019, 95% CI: 1.002–1.035) and indicated some risk when the PM_2.5_ concentration increased, although the result was not significant (RR = 1.007, 95% CI: 0.994–1.021). There was no difference in the effects of increased or decreased NO_2_ concentration between male and female outpatients with psoriasis (Supplementary Tables S3, S4 and Supplementary Figures S9–S11).

As shown in Supplementary Tables S5–S8 and Supplementary Figures S12–S17, outpatients with psoriasis aged 0–17 years old comprised the group most sensitive to the changed air pollutants concentrations, and the effects were similar to those of all the outpatients with psoriasis. Other age groups were influenced relatively little, but other interesting discoveries were found. Increasing NO_2_ by 20 μ*g*/*m*^3^ could increase the risk for outpatients with psoriasis who are older than 65 years (RR = 1.067, 95% CI: 1.035–1.099). The lag effect of PM_2.5_ was relatively stable in outpatients with psoriasis aged 40–64 years, which means the cumulative effects increased over time. In contrast to the results for all the included outpatients with psoriasis, SO_2_ was not risky to outpatients with psoriasis aged 0–17 years when concentrations were reduced.

## Discussion

Air pollution events occur frequently, posing an increasingly serious threat to human health. This threat is often reflected in the occurrence, deterioration, and recurrence of diseases and in the increase in outpatients with psoriasis. Based on the DLNM, this study showed that the effects of SO_2_ and PM_2.5_ on the number of outpatients with psoriasis were not significant at low concentrations but were not risky at high concentrations. At low concentrations, NO_2_ was not risky on the 1st day, but at high concentrations, its risk to outpatients with psoriasis increased; O_3_ displayed the opposite trend. Only NO_2_ manifested as a risk factor when the pollutants were increased. A stratified analysis showed that female subjects and outpatients with psoriasis aged 0–17 years were more sensitive to the change in air pollutants.

The mechanisms of air pollutants on psoriasis are difficult to monitor at an individual level, and a lag effect usually exists at the population level. Increased concentrations of PM_10_, SO_2_, and NO_2_ were associated with an increase in outpatient visits for post-adolescent acne in Xi'an, China (26). An increased risk of the Psoriasis Area and Severity Index was associated with increased concentrations of air pollutants, such as NO_2_ and particulate matter, 60 days prior to the increase in Verona, Italy (12). Increases in the PM_2.5_ and PM_10_ concentrations were associated with increases in patient visits for psoriasis in South Korea (27). Xu et al. (28) found that an increase of 10 μ*g*/*m*^3^ of O_3_ corresponds to an 0.87% increase in emergency room visits for all skin conditions, while PM_10_, SO_2_, and NO_2_ did not have substantial impacts.

Only NO_2_ increased the risk for outpatients with psoriasis when the concentrations of air pollutants increased, and the risk of O_3_ to outpatients with psoriasis increased when the concentration decreased in this study. Although increased concentrations of SO_2_, PM_2.5_, and O_3_ in outpatients with psoriasis showed no danger, the RRs or cumulative RRs increased following lag days, suggesting the effects of air pollutants on psoriasis are lagging and cumulative. Three reasons may explain this inconsistency. First, a different definition of low or high meteorological factors exists, and the range of meteorological factors varied with different study areas. Second, the day that patients came to the hospital did not necessarily represent the day of onset or worsening symptoms, as indicated by the lag effects. Third, people tend to stay indoors when the air quality is poor, thereby reducing their exposure to air pollutants.

Female outpatients with psoriasis were sensitive to changing concentrations of air pollutants, possibly because air pollutants could influence estrogen-regulated pathways (29), as suggested by the stronger impact of PM_10_ on post-adolescent acne in female subjects than in male subjects in Xi'an, China (26). Moreover, immune and skin barrier status may also play important roles (30–32) and could explain the phenomenon in which outpatients with psoriasis aged 0–17 years old were more sensitive to environmental factors than other ages. However, male subjects were more sensitive to changes in ADT and RH possibly due to more outdoor activities.

This study has limitations. First, although the study uses outpatient data from the two largest hospitals in Hefei, the data did not cover all patients in the city. These two hospitals are large-scale and leading-technology general hospitals in Hefei city, responsible for the most psoriasis diagnosis and treatment in the city. Therefore, on the premise of not being able to obtain the data on psoriasis in the whole city, we assume that the number of outpatients with psoriasis in these two hospitals can represent the data. Second, the estimation of the number of patients who do not consider themselves in need of medical treatment was not possible because these patients believe they are “slightly” affected by meteorological factors or air pollutants. Third, this study did not directly measure the actual exposure level of individual patients. As in similar studies, the mean or median exposure was used as the exposure level of the study population. Finally, the pathogenesis and treatment conditions of psoriasis are complicated, and more intricate studies are needed to explore the interaction of meteorological factors and air pollutants, with the immune status and genetic factors of patients.

## Conclusion

In conclusion, short-term air pollutant exposures were correlated with the occurrence of psoriasis in outpatients. Females, and outpatients with psoriasis aged 0–17 years old, were more sensitive to the changes in air pollutants. This finding suggests that patients and people at high risk of psoriasis should reduce their time outdoors and improve the quality of their protective gear when the air quality is poor.

## Data availability statement

The original contributions presented in the study are included in the article/Supplementary material, further inquiries can be directed to the corresponding authors.

## Ethics statement

The studies involving human participants were reviewed and approved by the School of Public Health, Anhui Medical University Research Ethics Committee. Written informed consent from the participants' legal guardian/next of kin was not required to participate in this study in accordance with the national legislation and the institutional requirements.

## Author contributions

TW participated in the conceptualization, analysis and interpretation of data, and manuscript preparation. YX contributed to conceptualization, data collection, data cleaning and discrepancy checks, and analysis and interpretation of data. XZ and NQ participated in data collection. SK and QF were involved in data cleaning and discrepancy checks. DY contributed to conceptualization. YF was involved in conceptualization, analytic strategy, and analysis and interpretation of data. All authors contributed to the article and approved the submitted version.
