# Supplementary material for: Short-term effects of air pollutants on outpatients with psoriasis in a Chinese city with a subtropical monsoon climate
Source: Front Public Health. 2022 Dec 22;10:1071263. doi: 10.3389/fpubh.2022.1071263 (PMC9817471; doi:10.3389/fpubh.2022.1071263)
Supplement: Supplementary file 1 [file Data_Sheet_1.docx]

**Supplementary materials**


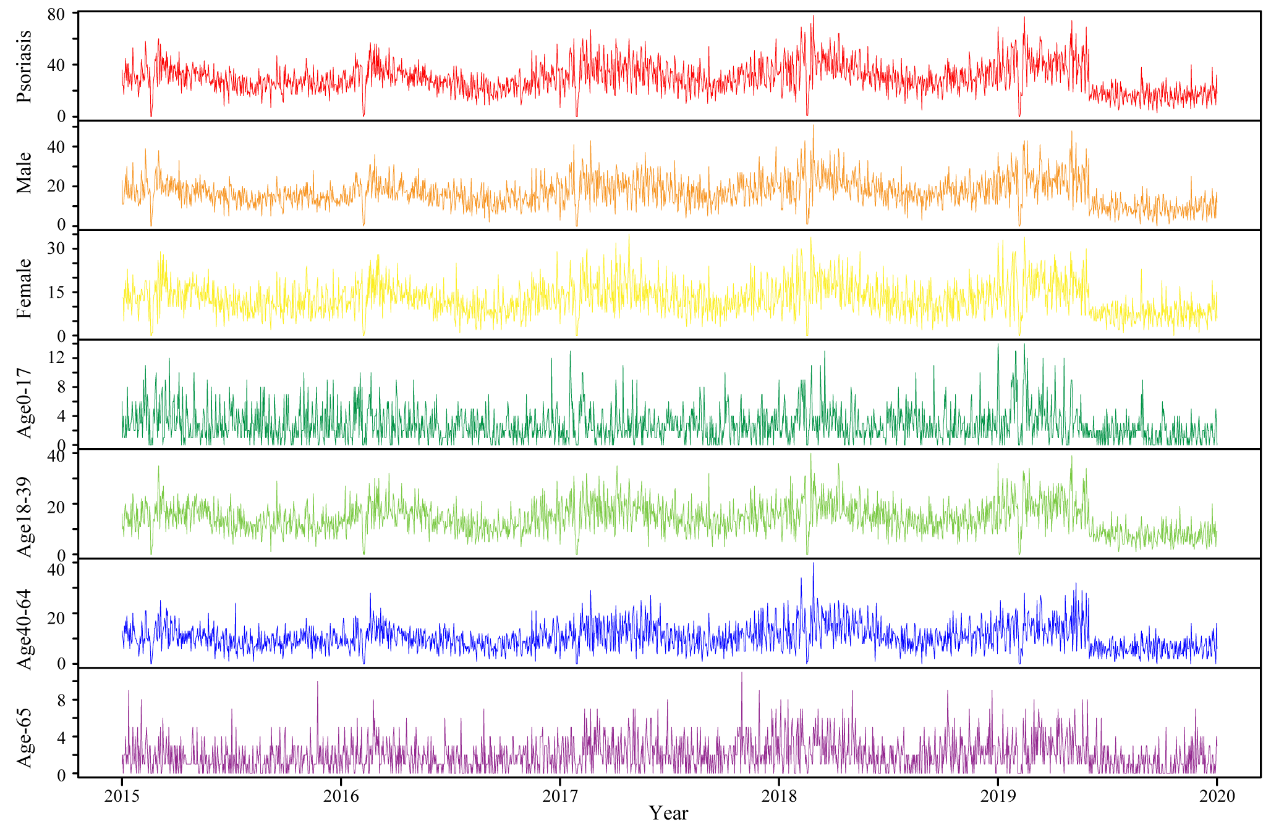


**Figure S1** Time series characteristics of the daily visits of outpatients with psoriasis in Hefei City from 2015 to 2019.


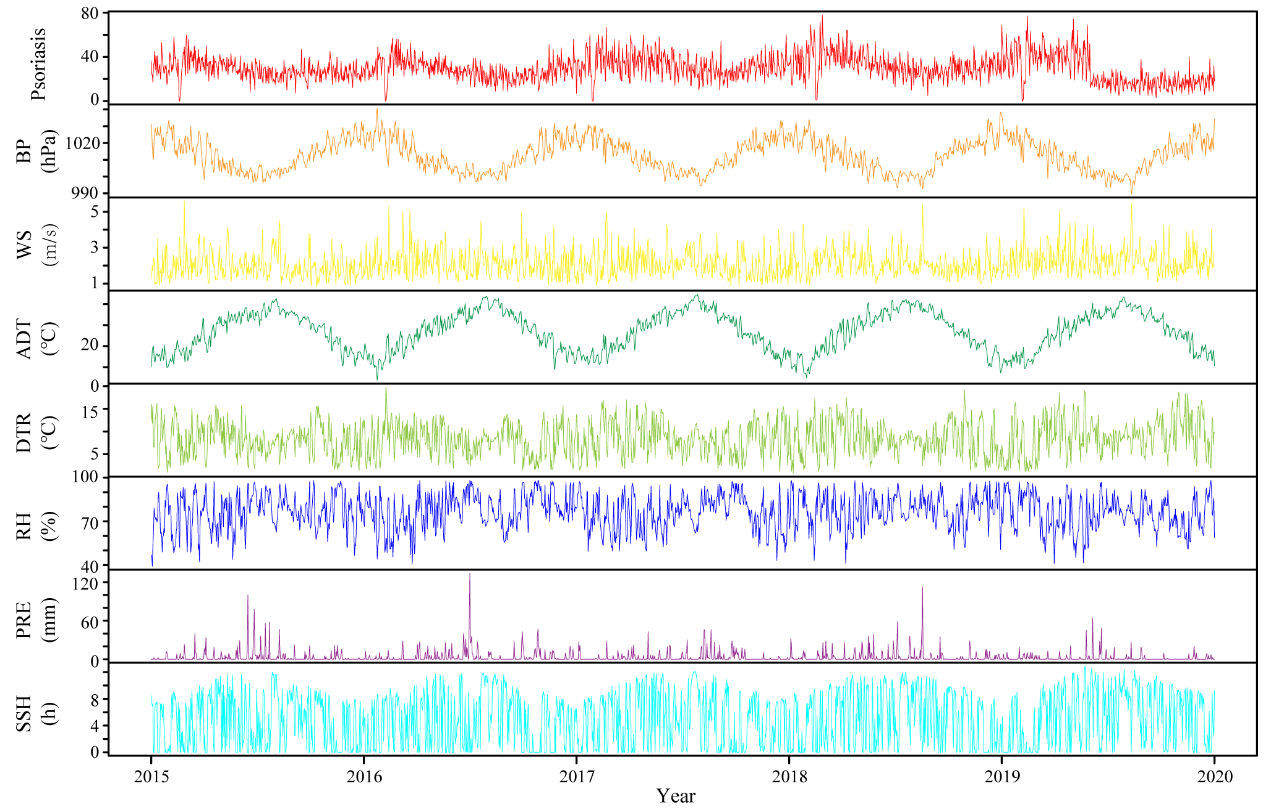


**Figure S2** Time series characteristics of meteorological factors in Hefei City from 2015 to 2019. [BP: barometric pressure. WS: wind speed. ADT: average daily temperature. DTR: diurnal temperature range. RH: relative humidity. PRE: precipitation. SSH: sunshine hour.]


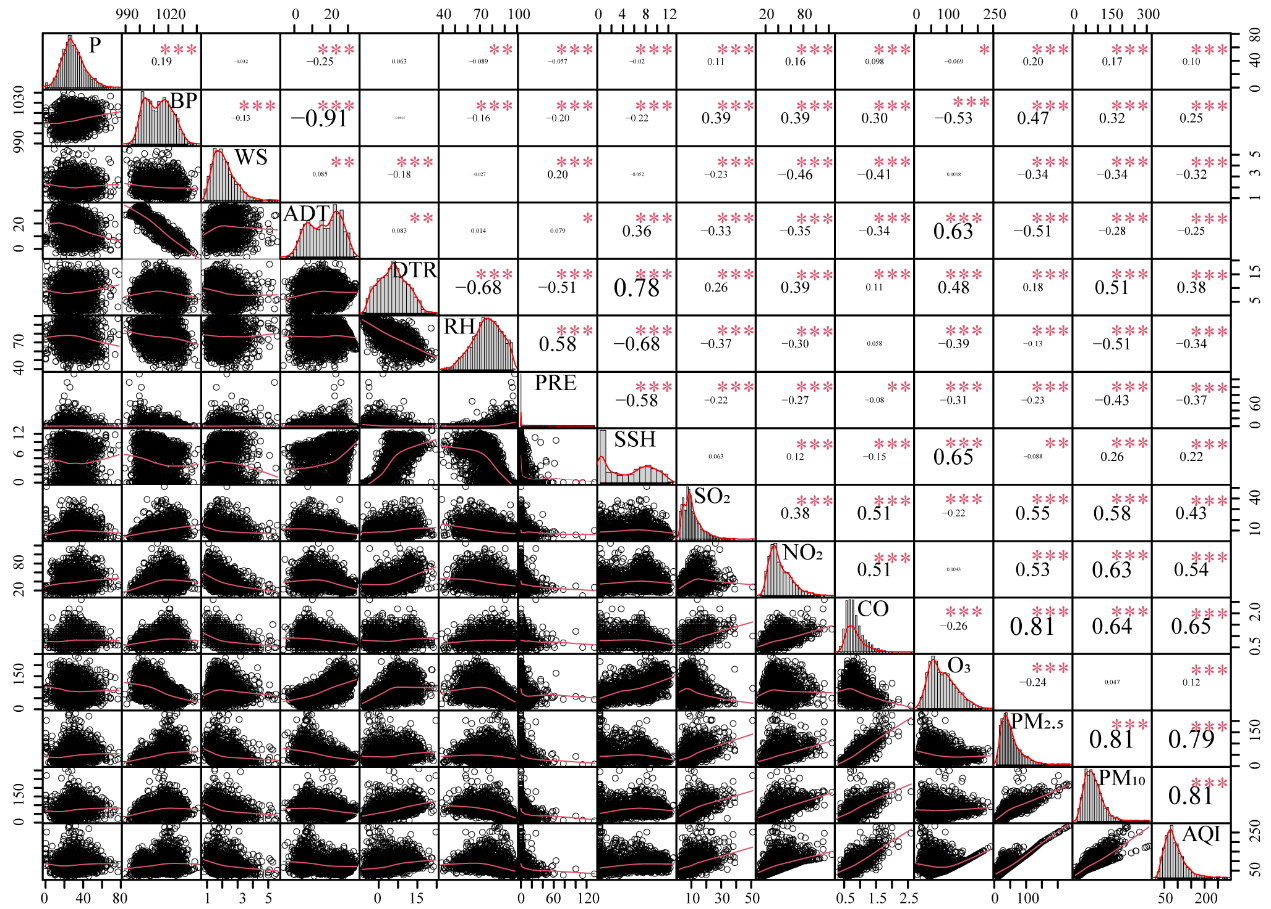


**Figure S3** Spearman correlation analysis of outpatients with psoriasis, meteorological factors and air pollutants in Hefei City from 2015 to 2019. [“***”: *P*<0.001, “**”: *P*<0.01, “*”: *P*<0.05. P: psoriasis. BP: barometric pressure. WS: wind speed. ADT: average daily temperature. DTR: diurnal temperature range. RH: relative humidity. PRE: precipitation. SSH: sunshine hour. SO_2_: sulfur dioxide. NO_2_: nitrogen dioxide. CO: carbon monoxide. O_3_: ozone. PM_2.5_: fine particulate matter. PM_10_: inhalable particulates. AQI: air quality index.]


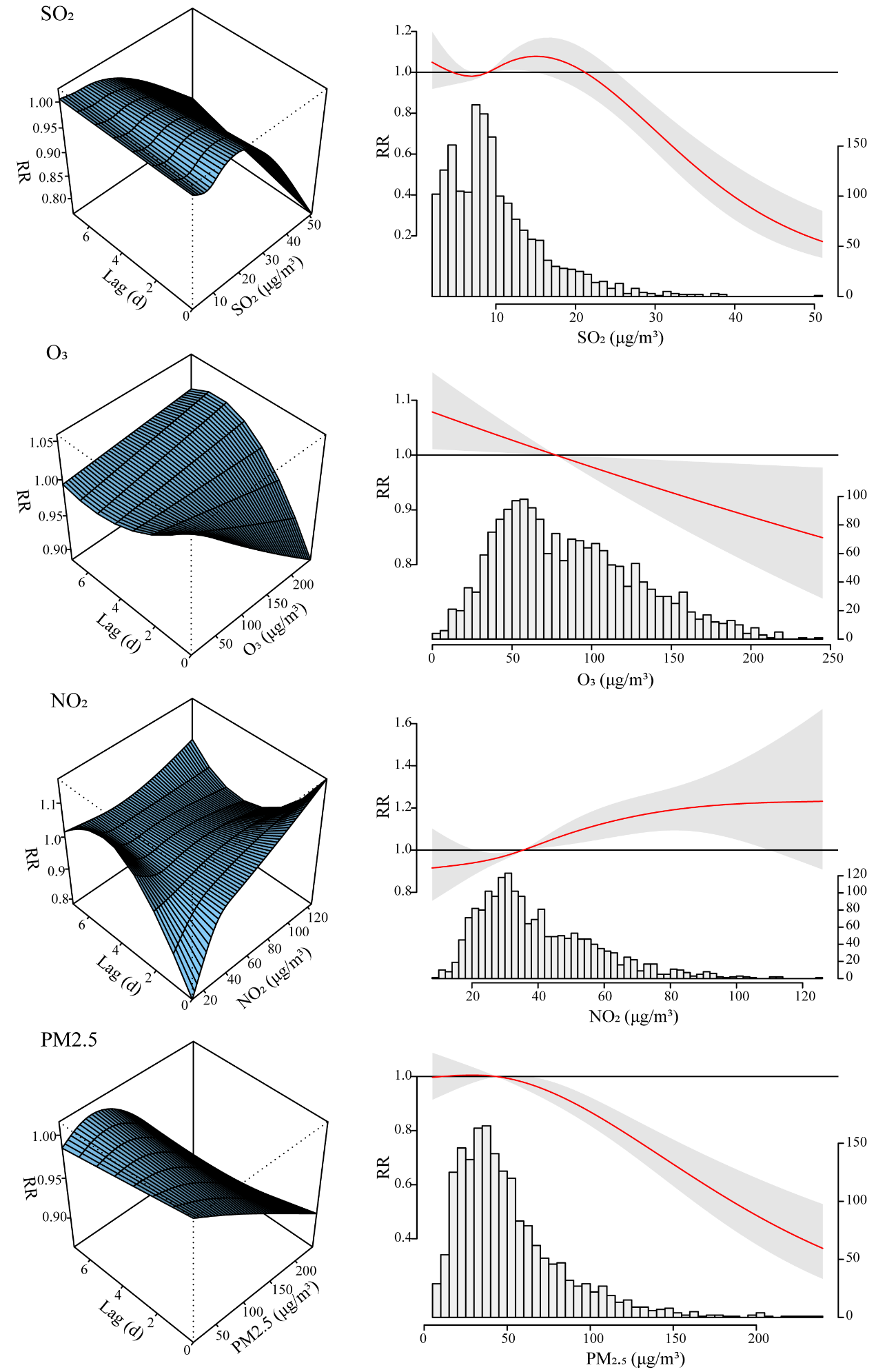


**Figure S4** Exposure-lag-response association of meteorological factors and air pollutants on outpatients with psoriasis.


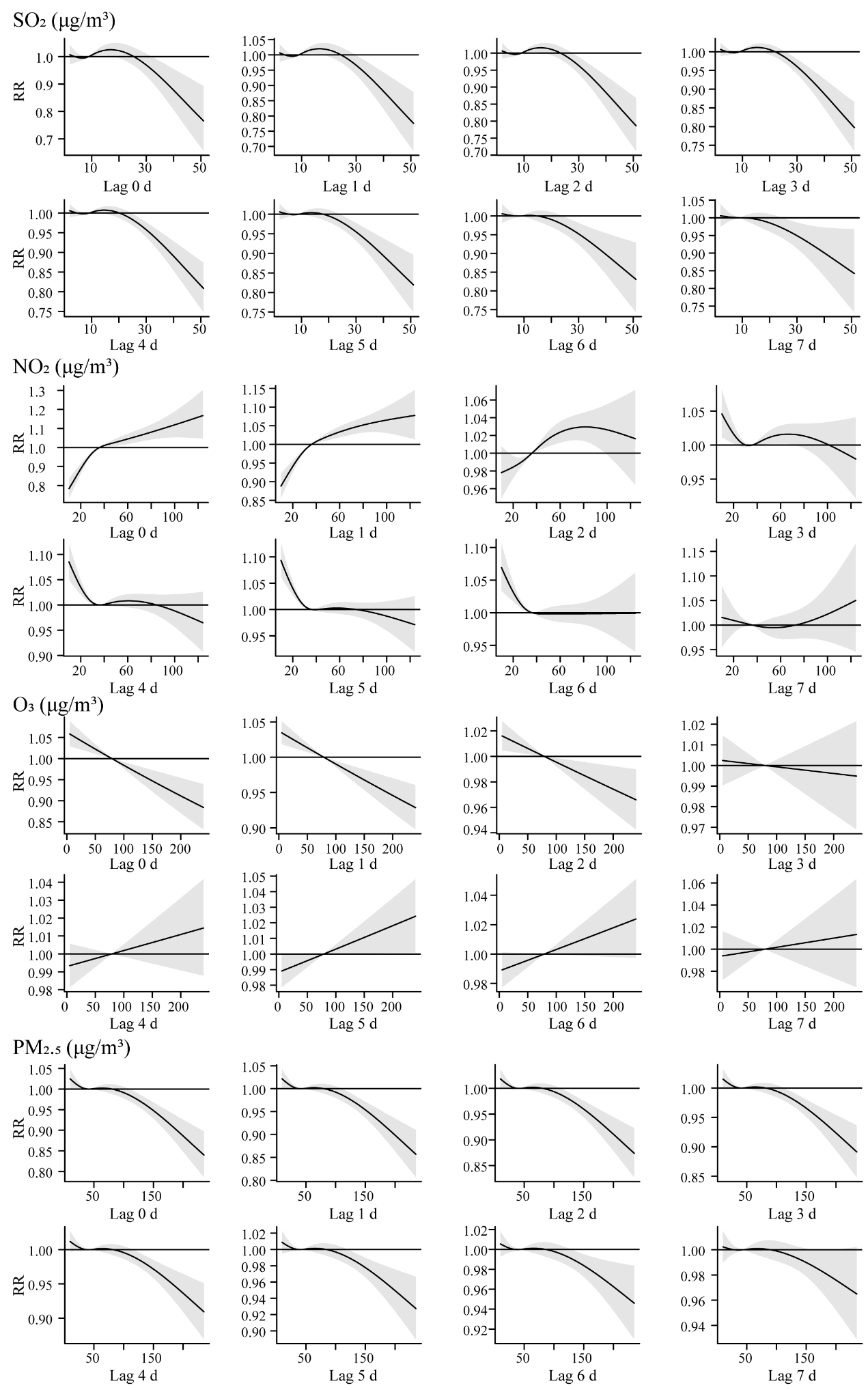


**Figure S5** Exposure-response association of air pollutants on outpatients with psoriasis at lag 0-7 days.


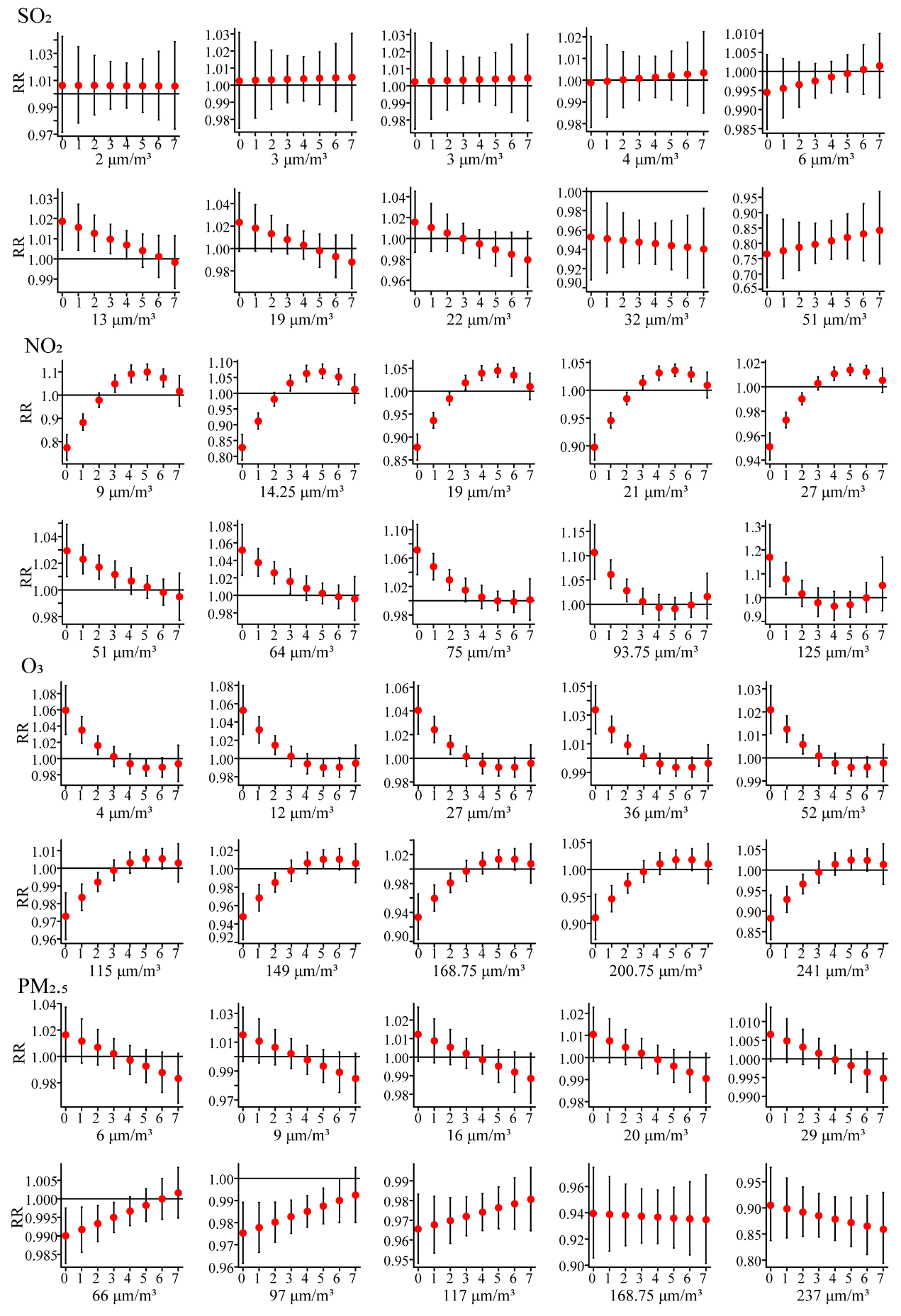


**Figure S6** Single-day lag-response association of air pollutants on outpatients with psoriasis. [At the 0%, 1%, 5%, 10%, 25%, 75%, 90%, 95%, 99%, 100% exposure.]


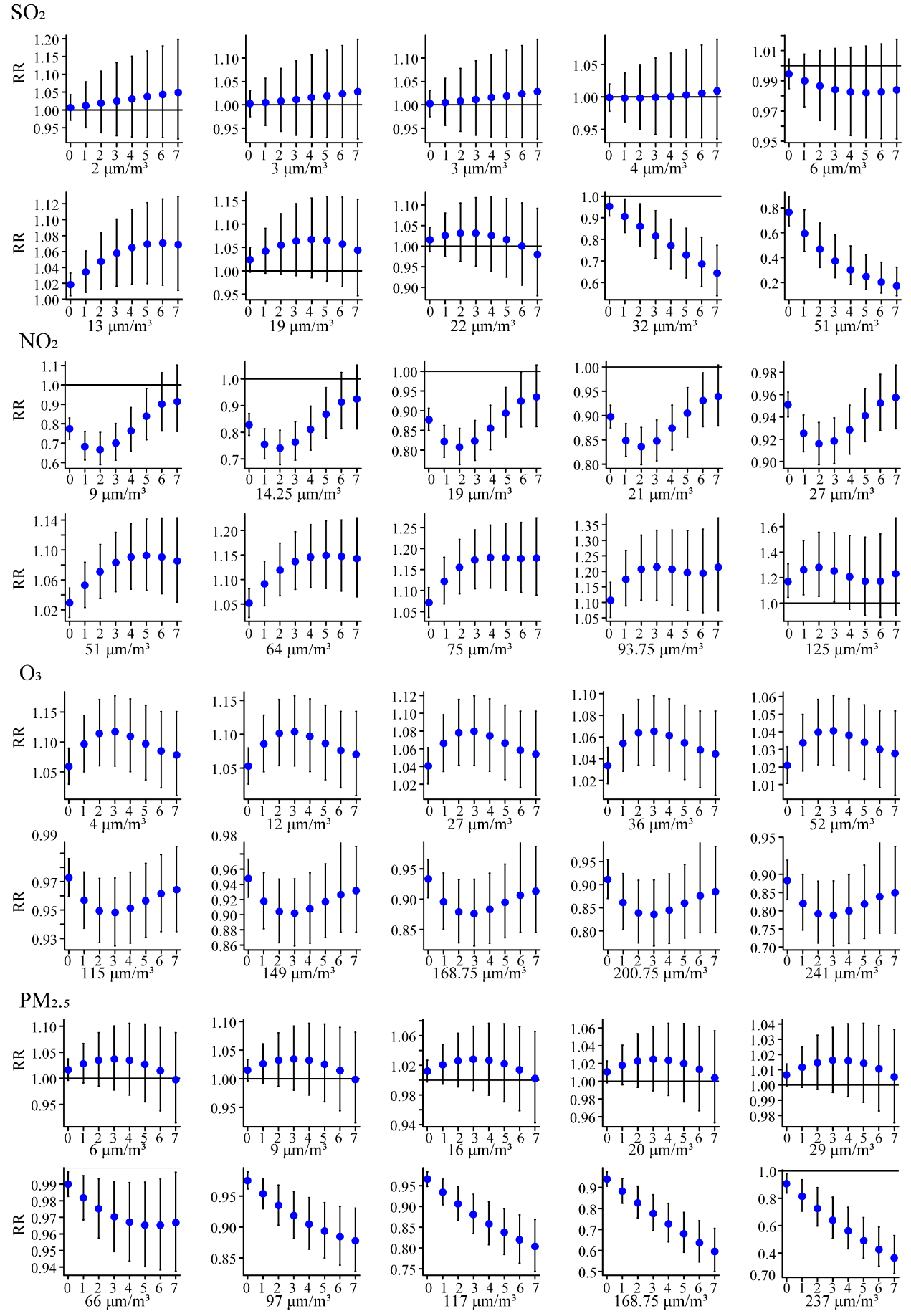


**Figure S7** Cumulative lag-response association of air pollutants on outpatients with psoriasis. [At the 0%, 1%, 5%, 10%, 25%, 75%, 90%, 95%, 99%, 100% exposure.]


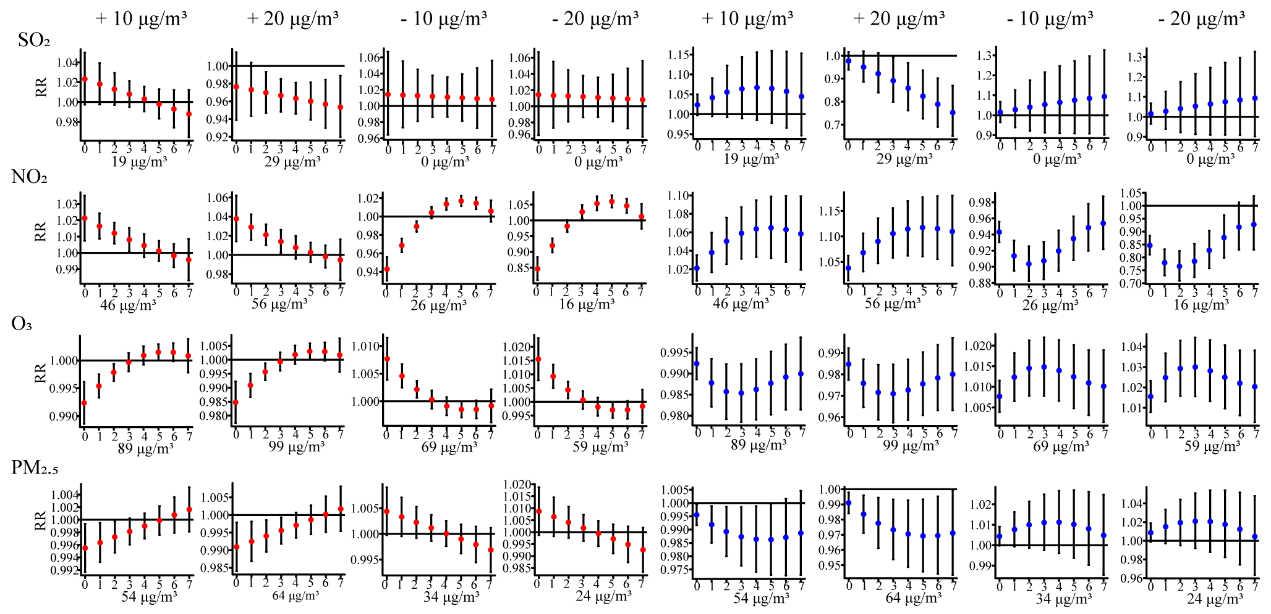


**Figure S8** Lag-response association of air pollutants on outpatients with psoriasis when changed 10 or 20 μg/m^3^. [Red graphs represent the single-day association and blue graphs represent the cumulative association.]


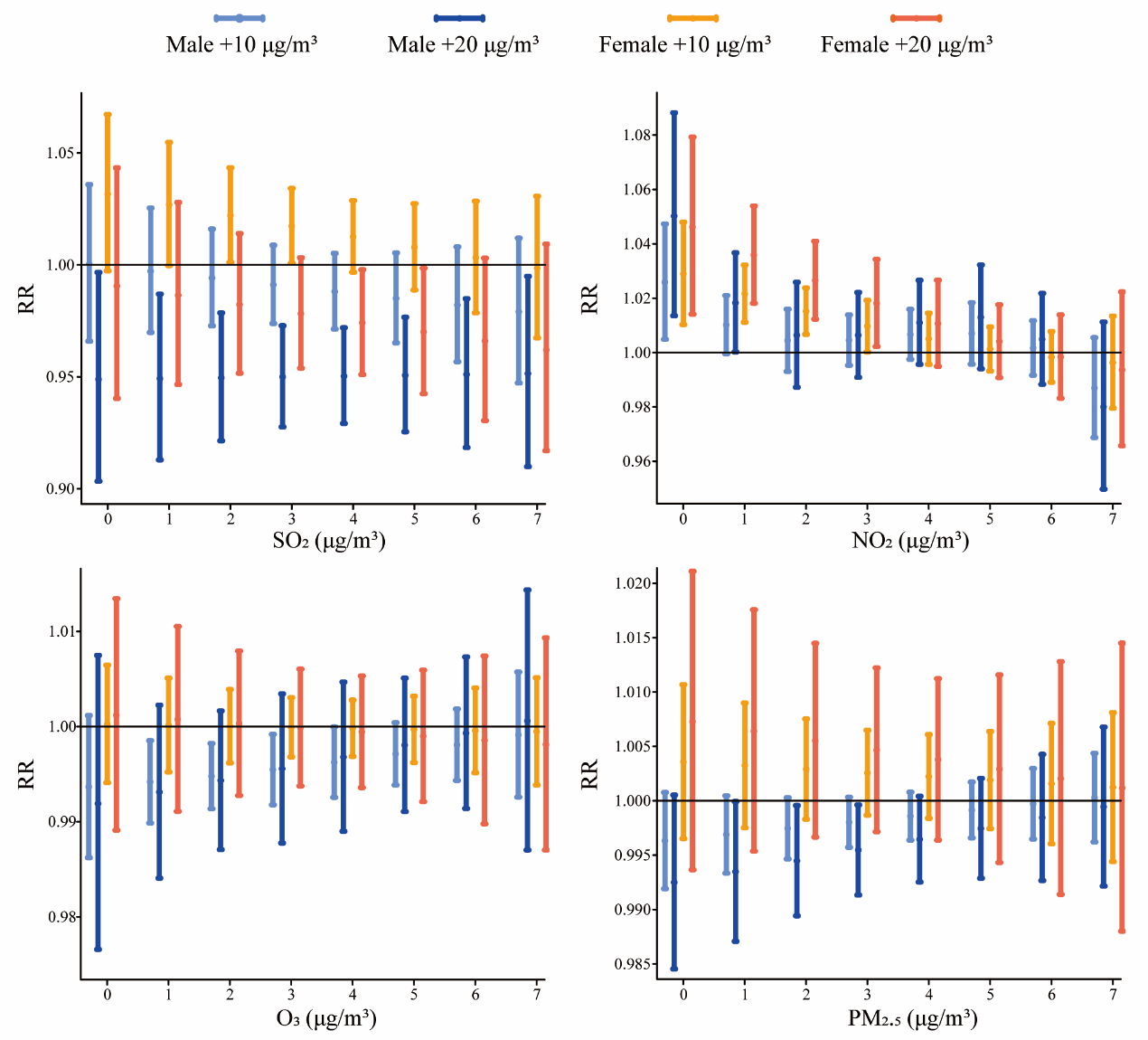


**Figure S9** Lag-response association of air pollutants on sex group when increased 10 or 20 μg/m^3^.


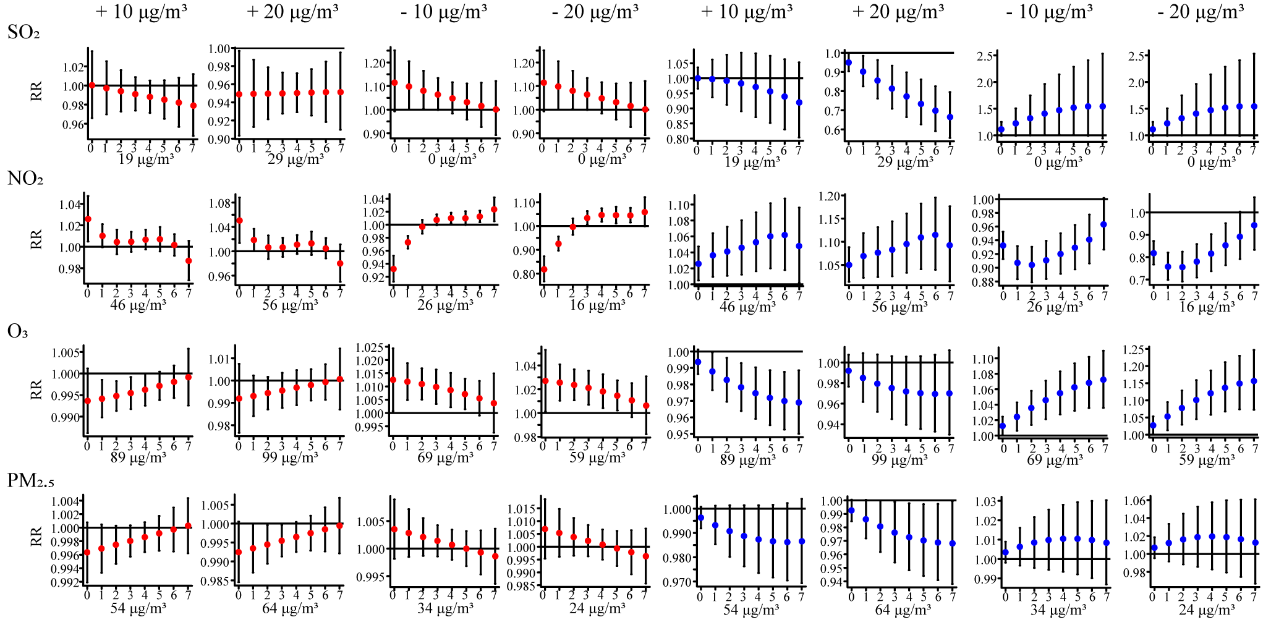


**Figure S10** Lag-response association of air pollutants on male outpatients with psoriasis when changed 10 or 20 μg/m^3^. [Red graphs represent the single-day association and blue graphs represent the cumulative association.]


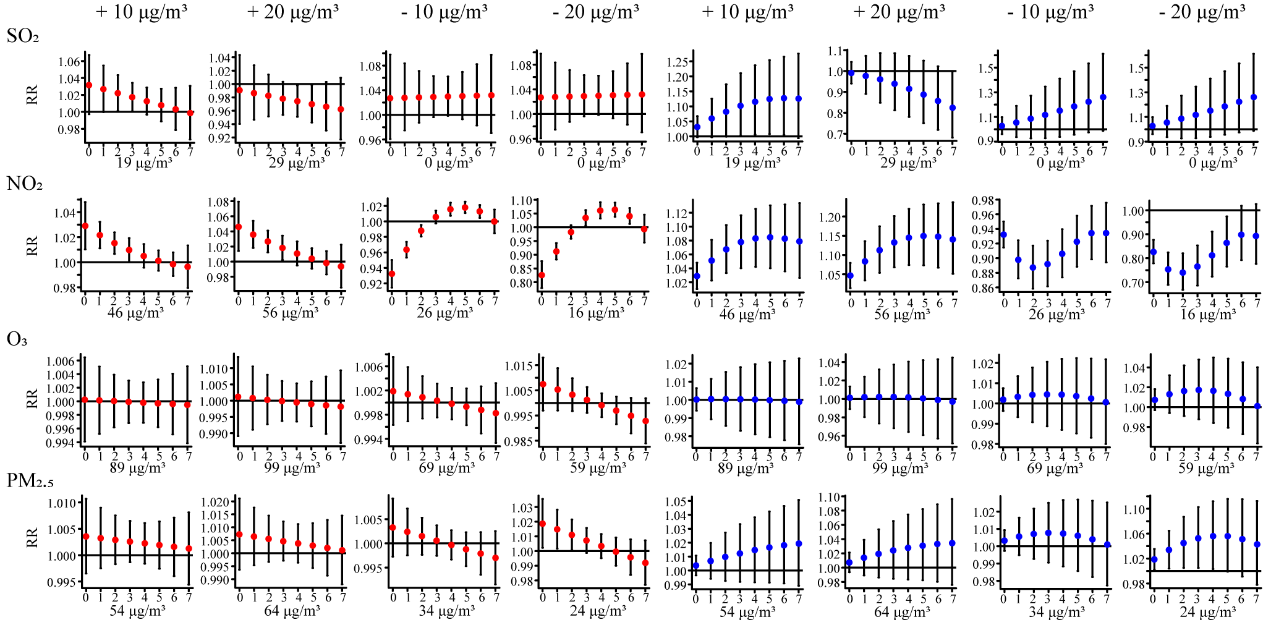


**Figure S11** Lag-response association of the air pollutants on female outpatients with psoriasis when changed 10 or 20 μg/m^3^. [Red graphs represent the single-day association and blue graphs represent the cumulative association.]


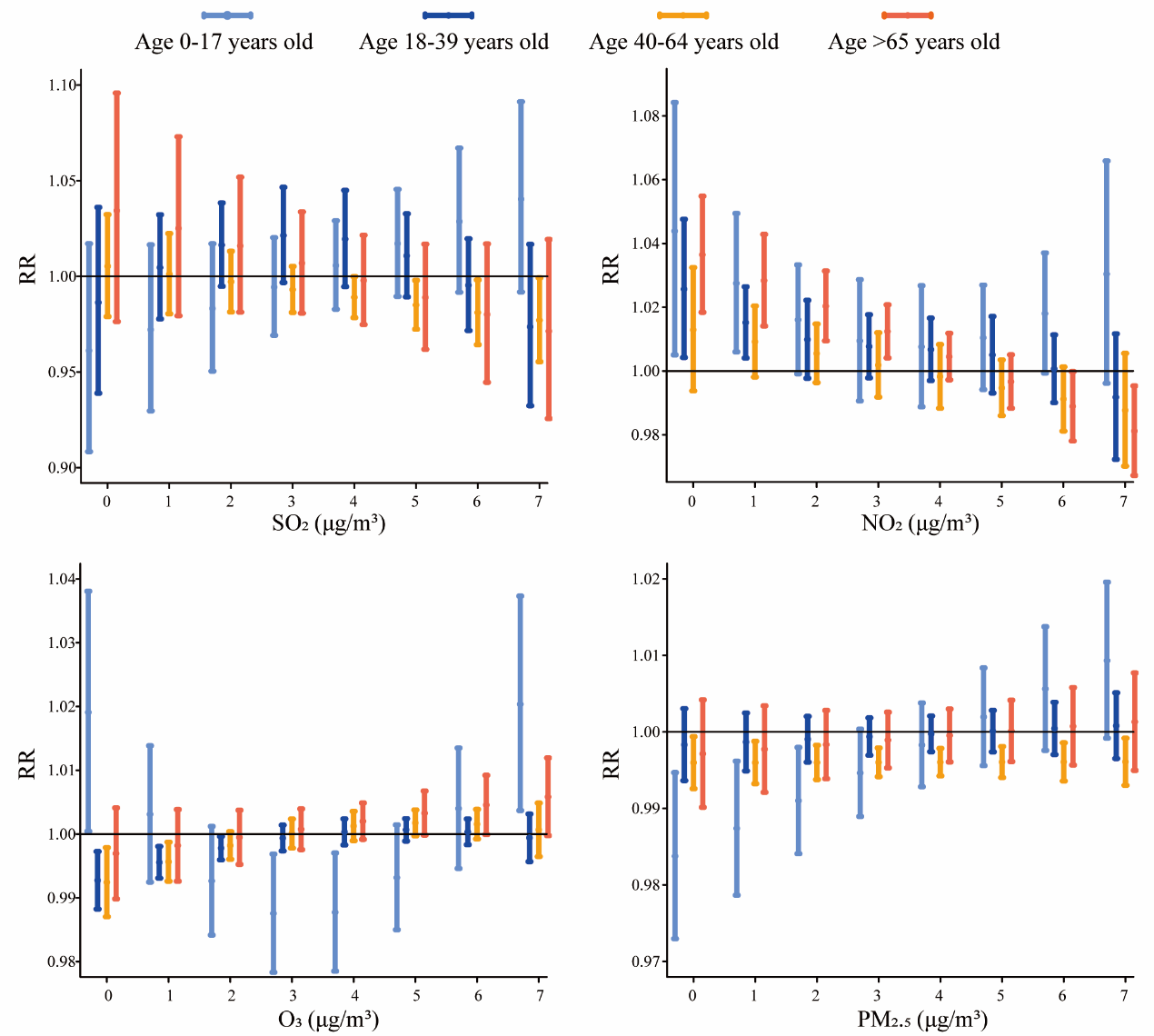


**Figure S12** Lag-response association of air pollutants on age group when increased 10 μg/m^3^.


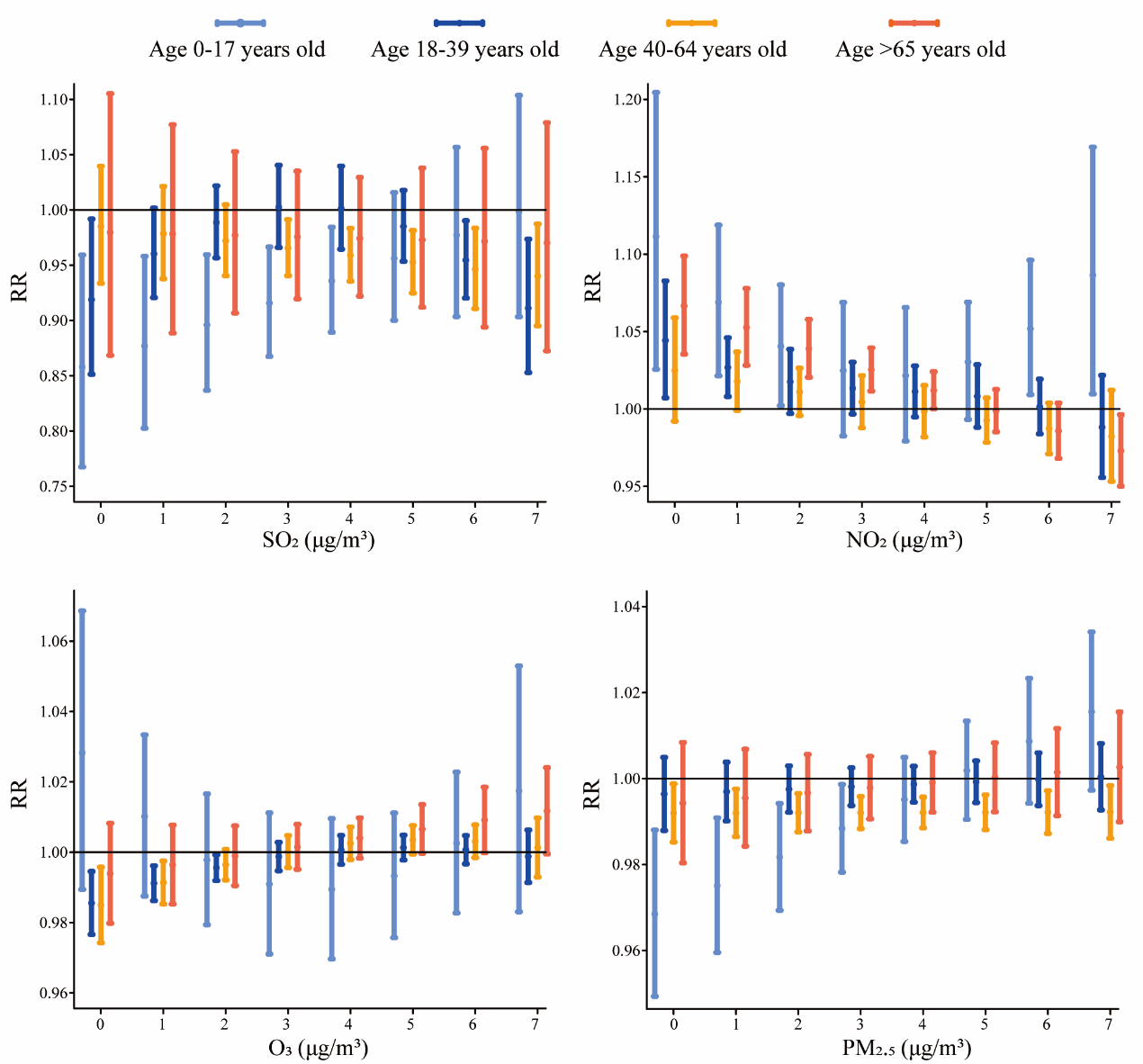


**Figure S13** Lag-response association of air pollutants on age group when increased 20 μg/m^3^.


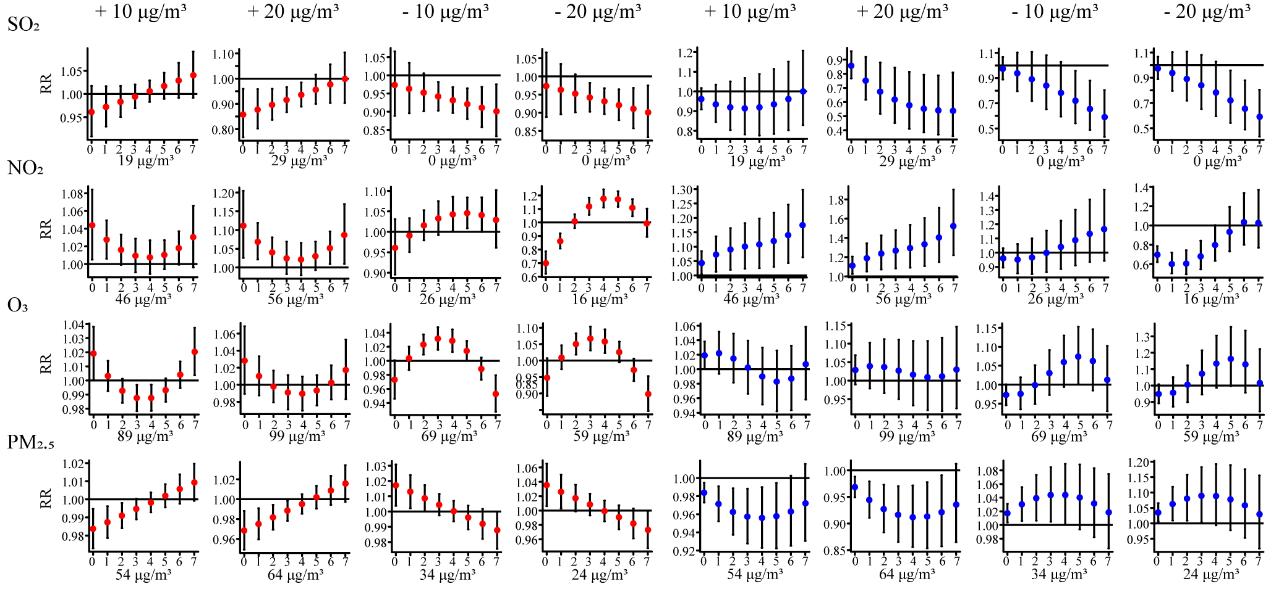


**Figure S14** Lag-response association of the air pollutants on outpatients with psoriasis aged 0-17 years old when changed 10 or 20 μg/m^3^. [Red graphs represent the single-day association and blue graphs represent the cumulative association.]


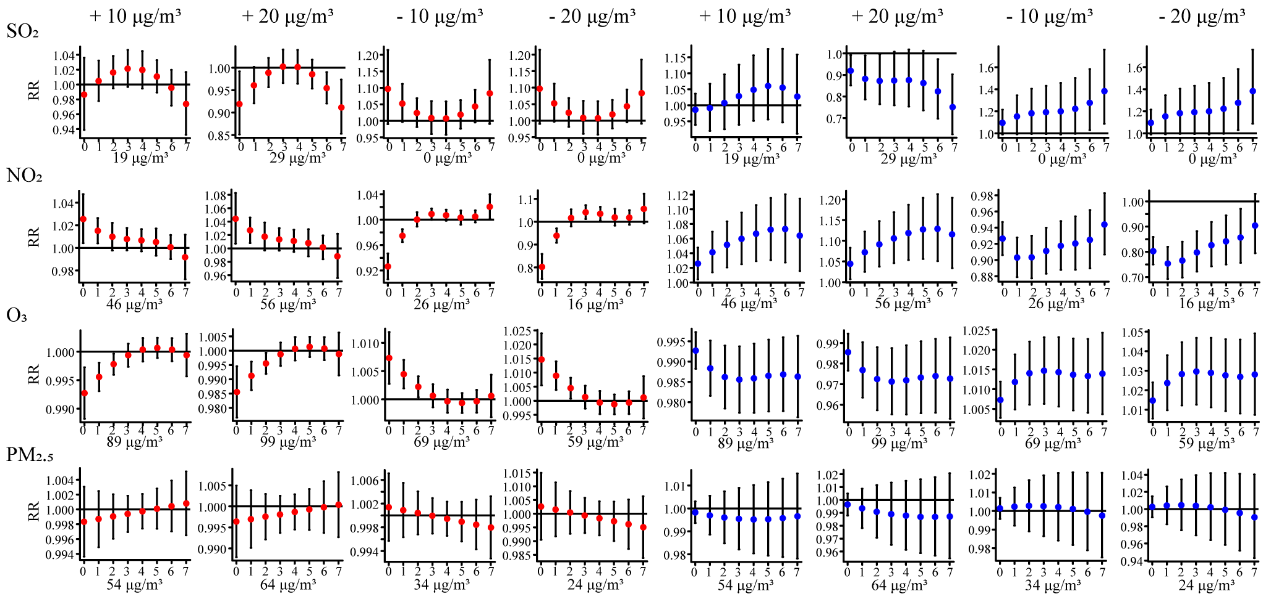


**Figure S15** Lag-response association of the air pollutants on outpatients with psoriasis aged 18-39 years old when changed 10 or 20 μg/m^3^. [Red graphs represent the single-day association and blue graphs represent the cumulative association.]


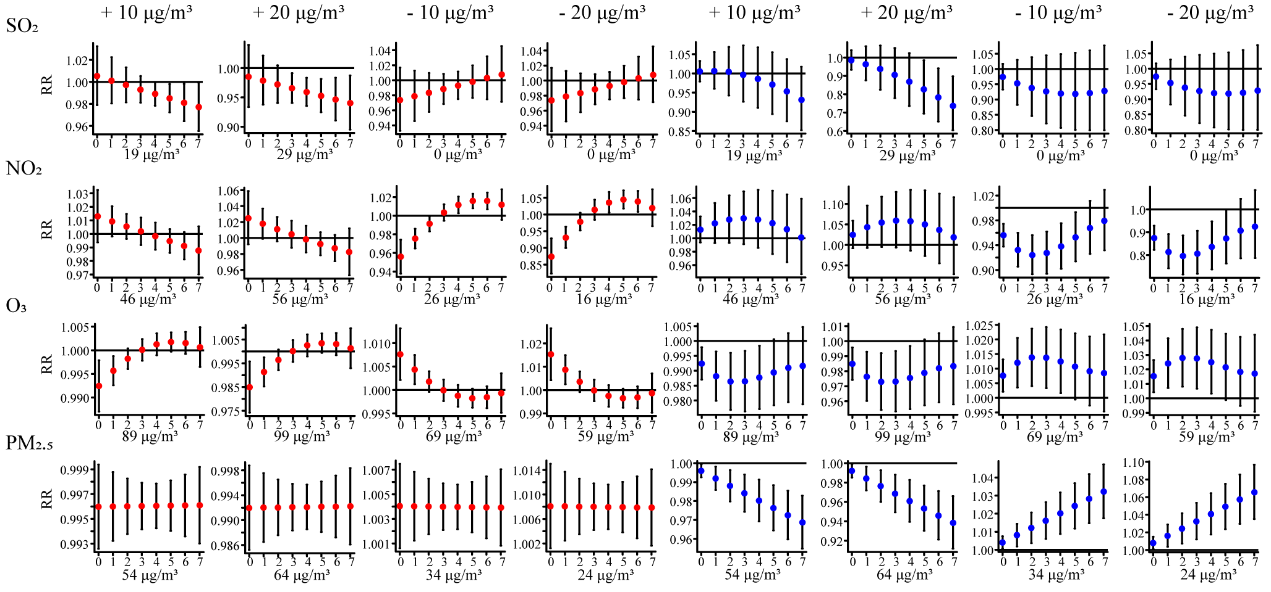


**Figure S16** Lag-response association of the air pollutants on outpatients with psoriasis aged 40-64 years old when changed 10 or 20 μg/m^3^. [Red graphs represent the single-day association and blue graphs represent the cumulative association.]


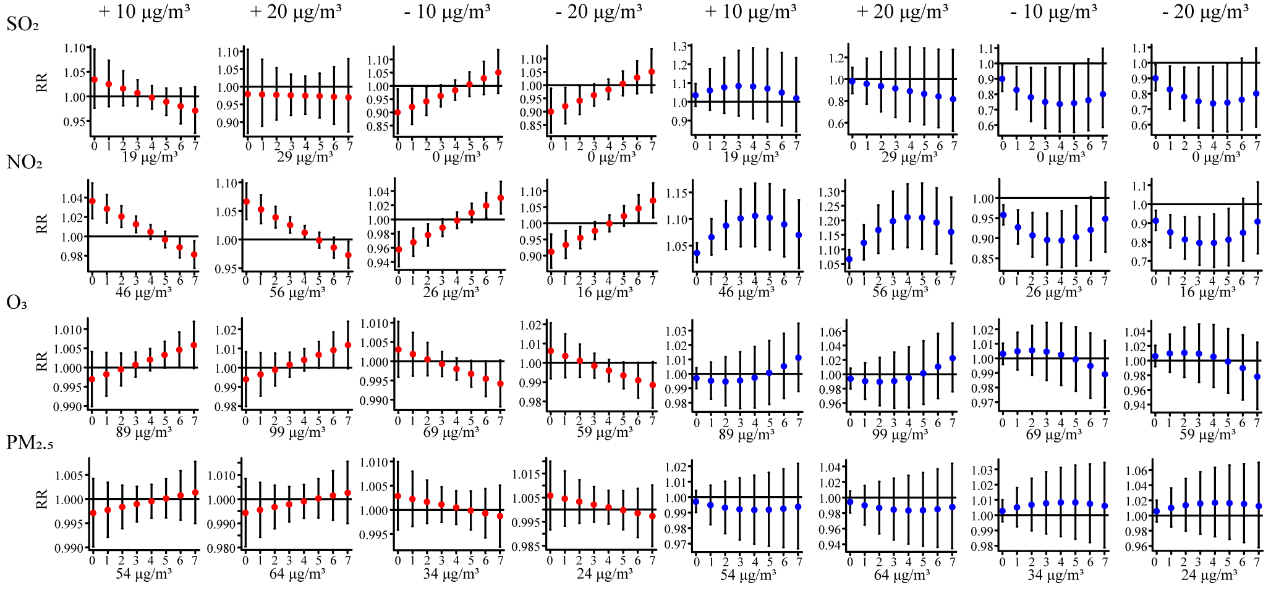


**Figure S17** Lag-response association of the air pollutants on outpatients with psoriasis elder than 65 years old when changed 10 or 20 μg/m^3^. [Red graphs represent the single-day association and blue graphs represent the cumulative association.]

**Table S1**  The maximum/minimum single-day and cumulative effects of air pollutants on outpatients with psoriasis.

|  | Low concentration | |  | High concentration | |
| --- | --- | --- | --- | --- | --- |
| Lag (d) | *RR* (95% *CI*) | Cumulative *RR* (95% *CI*) |  | *RR* (95% *CI*) | Cumulative *RR* (95% *CI*) |
| SO_2_ | 15 μg/m^3^ | |  | 51 μg/m^3^ | |
| 0 | 1.023(1.004, 1.043) ^a^ | 1.023(1.004, 1.043) ^a^ |  | 0.765(0.656, 0.893) ^a^ | 0.765(0.656, 0.893) ^a^ |
| 1 | 1.019(1.004, 1.035) ^a^ | 1.043(1.008, 1.079) ^a^ |  | 0.776(0.685, 0.879) ^a^ | 0.594(0.449, 0.784) ^a^ |
| 2 | 1.015(1.003, 1.028) ^a^ | 1.059(1.012, 1.109) ^a^ |  | 0.786(0.712, 0.868) ^a^ | 0.467(0.321, 0.678) ^a^ |
| 3 | 1.011(1.002, 1.021) ^a^ | 1.071(1.015, 1.131) ^a^ |  | 0.797(0.735, 0.865) ^a^ | 0.372(0.239, 0.581) ^a^ |
| 4 | 1.008(0.998, 1.017) | 1.079(1.017, 1.146) ^a^ |  | 0.808(0.748, 0.874) ^a^ | 0.301(0.183, 0.494) ^a^ |
| 5 | 1.004(0.993, 1.015) | 1.083(1.016, 1.155) ^a^ |  | 0.820(0.749, 0.896) ^a^ | 0.247(0.144, 0.421) ^a^ |
| 6 | 1.000(0.986, 1.014) | 1.083(1.011, 1.160) ^a^ |  | 0.831(0.743, 0.929) ^a^ | 0.205(0.116, 0.363) ^a^ |
| 7 | 0.996(0.978, 1.014) | 1.079(1.001, 1.162) ^a^ |  | 0.842(0.733, 0.968) ^a^ | 0.173(0.093, 0.322) ^a^ |
| NO_2_ | 9 μg/m^3^ | |  | 125 μg/m^3^ | |
| 0 | 0.773(0.721, 0.830) ^a^ | 0.773(0.721, 0.830) ^a^ |  | 1.170(1.046, 1.307) ^a^ | 1.170(1.046, 1.307) ^a^ |
| 1 | 0.883(0.849, 0.919) ^a^ | 0.683(0.614, 0.760) ^a^ |  | 1.078(1.013, 1.148) ^a^ | 1.261(1.066, 1.491) ^a^ |
| 2 | 0.978(0.947, 1.009) | 0.668(0.590, 0.756) ^a^ |  | 1.016(0.963, 1.072) | 1.281(1.054, 1.556) ^a^ |
| 3 | 1.049(1.012, 1.086) ^a^ | 0.700(0.612, 0.801) ^a^ |  | 0.979(0.920, 1.041) | 1.253(1.012, 1.553) ^a^ |
| 4 | 1.090(1.053, 1.130) ^a^ | 0.764(0.660, 0.884) ^a^ |  | 0.964(0.905, 1.026) | 1.208(0.954, 1.530) |
| 5 | 1.099(1.066, 1.133) ^a^ | 0.839(0.718, 0.981) ^a^ |  | 0.970(0.918, 1.026) | 1.172(0.906, 1.518) |
| 6 | 1.073(1.035, 1.113) ^a^ | 0.901(0.763, 1.063) |  | 0.999(0.939, 1.063) | 1.171(0.889, 1.542) |
| 7 | 1.016(0.953, 1.084) ^a^ | 0.915(0.760, 1.102) |  | 1.051(0.945, 1.170) | 1.231(0.908, 1.669) |
| O_3_ | 4 μg/m^3^ | |  | 241 μg/m^3^ | |
| 0 | 1.059(1.030, 1.090) ^a^ | 1.059(1.030, 1.090) ^a^ |  | 0.883(0.831, 0.939) ^a^ | 0.883(0.831, 0.939) ^a^ |
| 1 | 1.035(1.019, 1.052) ^a^ | 1.096(1.050, 1.145) ^a^ |  | 0.928(0.897, 0.960) ^a^ | 0.820(0.747, 0.900) ^a^ |
| 2 | 1.016(1.005, 1.028) ^a^ | 1.114(1.060, 1.171) ^a^ |  | 0.966(0.942, 0.990) ^a^ | 0.792(0.711, 0.881) ^a^ |
| 3 | 1.002(0.990, 1.015) | 1.117(1.060, 1.177) ^a^ |  | 0.995(0.969, 1.022) | 0.788(0.703, 0.882) ^a^ |
| 4 | 0.993(0.981, 1.006) | 1.109(1.050, 1.172) ^a^ |  | 1.015(0.988, 1.042) | 0.799(0.710, 0.899) ^a^ |
| 5 | 0.989(0.978, 1.000) | 1.097(1.037, 1.161) ^a^ |  | 1.024(1.001, 1.048) ^a^ | 0.818(0.724, 0.925) ^a^ |
| 6 | 0.989(0.977, 1.001) | 1.085(1.023, 1.151) ^a^ |  | 1.024(0.997, 1.051) | 0.838(0.738, 0.951) ^a^ |
| 7 | 0.994(0.972, 1.016) | 1.079(1.011, 1.151) ^a^ |  | 1.013(0.965, 1.064) | 0.849(0.738, 0.977) ^a^ |
| PM_2.5_ | 6 μg/m^3^ | |  | 237 μg/m^3^ | |
| 0 | 1.016(0.996, 1.037) | 1.016(0.996, 1.037) |  | 0.905(0.838, 0.977) ^a^ | 0.905(0.838, 0.977) ^a^ |
| 1 | 1.012(0.995, 1.028) | 1.028(0.991, 1.067) |  | 0.898(0.843, 0.957) ^a^ | 0.813(0.706, 0.935) ^a^ |
| 2 | 1.007(0.993, 1.020) | 1.035(0.985, 1.088) |  | 0.891(0.845, 0.940) ^a^ | 0.724(0.598, 0.877) ^a^ |
| 3 | 1.002(0.991, 1.013) | 1.037(0.978, 1.101) |  | 0.885(0.844, 0.927) ^a^ | 0.641(0.508, 0.808) ^a^ |
| 4 | 0.997(0.986, 1.008) | 1.035(0.968, 1.106) |  | 0.878(0.838, 0.921) ^a^ | 0.563(0.432, 0.734) ^a^ |
| 5 | 0.993(0.980, 1.005) | 1.027(0.955, 1.104) |  | 0.872(0.826, 0.920) ^a^ | 0.491(0.365, 0.659) ^a^ |
| 6 | 0.988(0.973, 1.003) | 1.014(0.938, 1.098) |  | 0.865(0.811, 0.924) ^a^ | 0.425(0.306, 0.590) ^a^ |
| 7 | 0.983(0.965, 1.002) | 0.997(0.914, 1.088) |  | 0.859(0.794, 0.929) ^a^ | 0.365(0.252, 0.528) ^a^ |

^a^: *P*<0.05.

**Table S2**  The single-day and cumulative effects of air pollutants on outpatients with psoriasis when changed by 10 or 20 μg/m^3^.

| Lag (d) | Single-day effects | | | |  | Cumulative effects | | | |
| --- | --- | --- | --- | --- | --- | --- | --- | --- | --- |
|  | 10 units increment | 20 units increment | 10 units decrement | 20 units decrement |  | 10 units increment | 20 units increment | 10 units decrement | 20 units decrement |
| SO_2_ (μg/m^3^) ^a^ |  |  |  |  |  |  |  |  |  |
| 0 | 1.023(0.997, 1.050) | 0.977(0.939, 1.015) | 1.014(0.964, 1.067) | - |  | 1.023(0.997, 1.050) | 0.977(0.939, 1.015) | 1.014(0.964, 1.067) |  |
| 1 | 1.018(0.998, 1.039) | 0.973(0.943, 1.004) | 1.013(0.973, 1.055) | - |  | 1.042(0.995, 1.091) | 0.950(0.886, 1.019) | 1.028(0.939, 1.126) |  |
| 2 | 1.013(0.997, 1.029) | 0.970(0.947, 0.993) ^b^ | 1.013(0.981, 1.045) | - |  | 1.055(0.993, 1.122) | 0.922(0.840, 1.012) | 1.041(0.922, 1.175) |  |
| 3 | 1.008(0.995, 1.021) | 0.967(0.948, 0.985) ^b^ | 1.012(0.986, 1.038) | - |  | 1.064(0.990, 1.144) | 0.891(0.799, 0.994) ^b^ | 1.053(0.913, 1.215) |  |
| 4 | 1.003(0.991, 1.016) | 0.963(0.946, 0.981) ^b^ | 1.011(0.987, 1.036) | - |  | 1.067(0.985, 1.156) | 0.859(0.762, 0.968) ^b^ | 1.064(0.909, 1.246) |  |
| 5 | 0.998(0.983, 1.013) | 0.960(0.939, 0.981) ^b^ | 1.010(0.981, 1.039) | - |  | 1.065(0.978, 1.159) | 0.824(0.726, 0.936) ^b^ | 1.075(0.909, 1.271) |  |
| 6 | 0.993(0.974, 1.012) | 0.957(0.930, 0.985) ^b^ | 1.009(0.973, 1.047) | - |  | 1.057(0.966, 1.157) | 0.789(0.690, 0.902) ^b^ | 1.085(0.908, 1.296) |  |
| 7 | 0.988(0.964, 1.012) | 0.954(0.920, 0.989) ^b^ | 1.008(0.962, 1.056) | - |  | 1.045(0.947, 1.153) | 0.752(0.651, 0.869) ^b^ | 1.093(0.902, 1.326) |  |
| NO_2_ (μg/m^3^) |  |  |  |  |  |  |  |  |  |
| 0 | 1.021(1.007, 1.035) ^b^ | 1.038(1.014, 1.062) ^b^ | 0.943(0.930, 0.956) ^b^ | 0.846(0.811, 0.883) ^b^ |  | 1.021(1.007, 1.035) ^b^ | 1.038(1.014, 1.062) ^b^ | 0.943(0.930, 0.956) ^b^ | 0.846(0.811, 0.883) ^b^ |
| 1 | 1.016(1.009, 1.024) ^b^ | 1.029(1.016, 1.042) ^b^ | 0.969(0.961, 0.976) ^b^ | 0.921(0.899, 0.943) ^b^ |  | 1.038(1.017, 1.060) ^b^ | 1.068(1.031, 1.106) ^b^ | 0.914(0.895, 0.933) ^b^ | 0.779(0.730, 0.831) ^b^ |
| 2 | 1.012(1.006, 1.019) ^b^ | 1.021(1.010, 1.032) ^b^ | 0.989(0.983, 0.995) ^b^ | 0.982(0.963, 1.001) |  | 1.050(1.026, 1.076) ^b^ | 1.090(1.047, 1.135) ^b^ | 0.904(0.882, 0.925) ^b^ | 0.765(0.709, 0.825) ^b^ |
| 3 | 1.008(1.001, 1.015) ^b^ | 1.014(1.002, 1.026) ^b^ | 1.004(0.998, 1.010) ^b^ | 1.027(1.006, 1.049) ^b^ |  | 1.059(1.031, 1.087) ^b^ | 1.106(1.058, 1.155) ^b^ | 0.907(0.884, 0.931) ^b^ | 0.786(0.724, 0.852) ^b^ |
| 4 | 1.004(0.997, 1.012) | 1.008(0.996, 1.020) | 1.013(1.007, 1.020) ^b^ | 1.053(1.032, 1.076) ^b^ |  | 1.064(1.033, 1.095) ^b^ | 1.114(1.062, 1.169) ^b^ | 0.920(0.895, 0.945) ^b^ | 0.827(0.758, 0.903) ^b^ |
| 5 | 1.001(0.995, 1.007) | 1.003(0.993, 1.013) | 1.017(1.011, 1.022) ^b^ | 1.060(1.041, 1.079) ^b^ |  | 1.065(1.032, 1.099) ^b^ | 1.117(1.061, 1.177) ^b^ | 0.935(0.908, 0.963) ^b^ | 0.877(0.798, 0.963) ^b^ |
| 6 | 0.998(0.991, 1.005) | 0.998(0.987, 1.010) | 1.014(1.008, 1.021) ^b^ | 1.046(1.024, 1.068) ^b^ |  | 1.063(1.028, 1.099) ^b^ | 1.115(1.055, 1.179) ^b^ | 0.948(0.920, 0.978) ^b^ | 0.917(0.830, 1.013) |
| 7 | 0.996(0.983, 1.009) | 0.995(0.974, 1.016) | 1.006(0.994, 1.018) | 1.012(0.974, 1.052) ^b^ |  | 1.058(1.019, 1.099) ^b^ | 1.109(1.043, 1.180) ^b^ | 0.954(0.922, 0.987) ^b^ | 0.928(0.830, 1.037) |
| O_3_ (μg/m^3^) |  |  |  |  |  |  |  |  |  |
| 0 | 0.992(0.989, 0.996) ^b^ | 0.985(0.977, 0.992) ^b^ | 1.008(1.004, 1.012) ^b^ | 1.015(1.008, 1.023) ^b^ |  | 0.992(0.989, 0.996) ^b^ | 0.985(0.977, 0.992) ^b^ | 1.008(1.004, 1.012) ^b^ | 1.015(1.008, 1.023) ^b^ |
| 1 | 0.995(0.993, 0.998) ^b^ | 0.991(0.987, 0.995) ^b^ | 1.005(1.002, 1.007) ^b^ | 1.009(1.005, 1.014) ^b^ |  | 0.988(0.982, 0.994) ^b^ | 0.976(0.965, 0.987) ^b^ | 1.012(1.007, 1.018) ^b^ | 1.025(1.013, 1.037) ^b^ |
| 2 | 0.998(0.996, 0.999) ^b^ | 0.996(0.993, 0.999) ^b^ | 1.002(1.001, 1.004) ^b^ | 1.004(1.001, 1.007) ^b^ |  | 0.986(0.979, 0.992) ^b^ | 0.972(0.959, 0.985) ^b^ | 1.015(1.008, 1.021) ^b^ | 1.029(1.016, 1.043) ^b^ |
| 3 | 1.000(0.998, 1.001) | 0.999(0.996, 1.003) | 1.000(0.999, 1.002) | 1.001(0.997, 1.004) |  | 0.985(0.978, 0.992) ^b^ | 0.971(0.957, 0.985) ^b^ | 1.015(1.008, 1.022) ^b^ | 1.030(1.016, 1.044) ^b^ |
| 4 | 1.001(0.999, 1.003) | 1.002(0.998, 1.005) | 0.999(0.997, 1.001) | 0.998(0.995, 1.002) |  | 0.986(0.979, 0.993) ^b^ | 0.973(0.959, 0.987) ^b^ | 1.014(1.007, 1.021) ^b^ | 1.028(1.013, 1.043) ^b^ |
| 5 | 1.001(1.000, 1.003) ^b^ | 1.003(1.000, 1.006) ^b^ | 0.999(0.997, 1.000) ^b^ | 0.997(0.994, 1.000) ^b^ |  | 0.988(0.980, 0.995) ^b^ | 0.976(0.961, 0.990) ^b^ | 1.012(1.005, 1.020) ^b^ | 1.025(1.010, 1.041) ^b^ |
| 6 | 1.001(1.000, 1.003) | 1.003(1.000, 1.006) | 0.999(0.997, 1.000) | 0.997(0.994, 1.000) |  | 0.989(0.981, 0.997) ^b^ | 0.978(0.963, 0.994) ^b^ | 1.011(1.003, 1.019) ^b^ | 1.022(1.006, 1.038) ^b^ |
| 7 | 1.001(0.998, 1.004) | 1.002(0.996, 1.008) | 0.999(0.996, 1.002) | 0.998(0.992, 1.004) |  | 0.990(0.981, 0.999) ^b^ | 0.980(0.963, 0.997) ^b^ | 1.010(1.001, 1.019) ^b^ | 1.020(1.003, 1.038) ^b^ |
| PM_2.5_ (μg/m^3^) |  |  |  |  |  |  |  |  |  |
| 0 | 0.996(0.992, 0.999) ^b^ | 0.991(0.984, 0.998) ^b^ | 1.004(1.000, 1.009) | 1.009(0.999, 1.019) |  | 0.996(0.992, 0.999) ^b^ | 0.991(0.984, 0.998) ^b^ | 1.004(1.000, 1.009) | 1.009(0.999, 1.019) |
| 1 | 0.996(0.993, 1.000) ^b^ | 0.992(0.987, 0.998) ^b^ | 1.003(1.000, 1.007) | 1.006(0.998, 1.015) |  | 0.992(0.985, 0.999) ^b^ | 0.984(0.971, 0.996) ^b^ | 1.008(0.999, 1.016) | 1.015(0.997, 1.033) |
| 2 | 0.997(0.995, 1.000) ^b^ | 0.994(0.990, 0.999) ^b^ | 1.002(0.999, 1.005) | 1.004(0.998, 1.011) |  | 0.989(0.980, 0.999) ^b^ | 0.978(0.961, 0.994) ^b^ | 1.010(0.999, 1.021) | 1.019(0.995, 1.044) |
| 3 | 0.998(0.996, 1.000) | 0.996(0.992, 0.999) ^b^ | 1.001(0.999, 1.004) | 1.002(0.996, 1.007) |  | 0.987(0.976, 0.998) ^b^ | 0.973(0.954, 0.993) ^b^ | 1.011(0.998, 1.025) | 1.021(0.992, 1.051) |
| 4 | 0.999(0.997, 1.001) | 0.997(0.994, 1.001) | 1.000(0.998, 1.003) | 0.999(0.994, 1.005) |  | 0.986(0.974, 0.999) ^b^ | 0.970(0.949, 0.993) ^b^ | 1.011(0.996, 1.027) | 1.020(0.988, 1.054) |
| 5 | 1.000(0.998, 1.002) | 0.999(0.995, 1.003) | 0.999(0.996, 1.002) | 0.997(0.991, 1.003) |  | 0.986(0.973, 1.000) ^b^ | 0.969(0.946, 0.993) ^b^ | 1.010(0.994, 1.027) | 1.018(0.982, 1.054) |
| 6 | 1.001(0.998, 1.004) | 1.000(0.995, 1.005) | 0.998(0.994, 1.001) | 0.995(0.987, 1.002) |  | 0.987(0.973, 1.002) | 0.969(0.944, 0.995) ^b^ | 1.008(0.990, 1.026) | 1.012(0.974, 1.052) |
| 7 | 1.002(0.998, 1.005) | 1.002(0.995, 1.008) | 0.997(0.993, 1.001) | 0.993(0.983, 1.002) |  | 0.989(0.973, 1.005) | 0.971(0.944, 0.999) ^b^ | 1.005(0.985, 1.025) | 1.005(0.963, 1.048) |

^a^: The median concentration of SO_2_ is 9 μg/m^3^, so defined 0 μg/m^3^ as the concentration reduced by 10 μg/m^3^, there is no the concentration reduced by 20 μg/m^3^.

^b^: *P*<0.05.

**Table S3**  The single-day and cumulative effects of air pollutants on male outpatients with psoriasis when changed by 10 or 20 μg/m^3^.

| Lag (d) | Single-day effects | | | |  | Cumulative effects | | | |
| --- | --- | --- | --- | --- | --- | --- | --- | --- | --- |
|  | 10 units increment | 20 units increment | 10 units decrement | 20 units decrement |  | 10 units increment | 20 units increment | 10 units decrement | 20 units decrement |
| SO_2_ (μg/m^3^) ^a^ |  |  |  |  |  | 1.000(0.966, 1.036) | 0.949(0.903, 0.997) ^a^ | 1.115(0.993, 1.251) | - |
| 0 | 1.000(0.966, 1.036) | 0.949(0.903, 0.997) ^b^ | 1.115(0.993, 1.251) | - |  | 0.997(0.937, 1.062) | 0.901(0.825, 0.983) ^b^ | 1.223(0.993, 1.506) | - |
| 1 | 0.997(0.970, 1.025) | 0.949(0.913, 0.987) ^b^ | 1.097(1.000, 1.205) | - |  | 0.992(0.912, 1.078) | 0.855(0.761, 0.961) ^b^ | 1.322(0.999, 1.749) | - |
| 2 | 0.994(0.973, 1.016) | 0.950(0.921, 0.979) ^b^ | 1.081(1.003, 1.165) ^b^ | - |  | 0.983(0.891, 1.085) | 0.812(0.708, 0.932) ^b^ | 1.407(1.008, 1.964) ^b^ | - |
| 3 | 0.991(0.974, 1.009) | 0.950(0.928, 0.973) ^b^ | 1.064(0.999, 1.134) | - |  | 0.971(0.871, 1.083) | 0.772(0.664, 0.897) ^b^ | 1.474(1.014, 2.142) ^b^ | - |
| 4 | 0.988(0.971, 1.005) | 0.950(0.929, 0.972) ^b^ | 1.048(0.984, 1.116) | - |  | 0.957(0.852, 1.074) | 0.734(0.626, 0.860) ^b^ | 1.521(1.012, 2.285) ^b^ | - |
| 5 | 0.985(0.965, 1.005) | 0.951(0.925, 0.977) ^b^ | 1.032(0.958, 1.111) | - |  | 0.939(0.830, 1.063) | 0.698(0.591, 0.825) ^b^ | 1.545(0.991, 2.407) | - |
| 6 | 0.982(0.957, 1.008) | 0.951(0.918, 0.985) ^b^ | 1.016(0.926, 1.114) | - |  | 0.920(0.804, 1.053) | 0.664(0.555, 0.795) ^b^ | 1.545(0.943, 2.530) | - |
| 7 | 0.979(0.947, 1.012) | 0.951(0.910, 0.995) ^b^ | 1.000(0.892, 1.121) | - |  |  |  |  |  |
| NO_2_ (μg/m^3^) |  |  |  |  |  | 1.026(1.005, 1.047) ^b^ | 1.050(1.014, 1.088) ^b^ | 0.932(0.913, 0.952) ^b^ | 0.818(0.767, 0.872) ^b^ |
| 0 | 1.026(1.005, 1.047) ^b^ | 1.050(1.014, 1.088) ^b^ | 0.932(0.913, 0.952) ^b^ | 0.818(0.767, 0.872) ^b^ |  | 1.036(1.009, 1.064) ^b^ | 1.069(1.022, 1.119) ^b^ | 0.907(0.883, 0.932) ^b^ | 0.757(0.699, 0.820) ^b^ |
| 1 | 1.010(1.000, 1.021) | 1.018(1.000, 1.037) ^b^ | 0.973(0.963, 0.983) ^b^ | 0.926(0.897, 0.956) ^b^ |  | 1.041(1.011, 1.072) ^b^ | 1.076(1.024, 1.131) ^b^ | 0.904(0.879, 0.930) ^b^ | 0.755(0.691, 0.824) ^b^ |
| 2 | 1.004(0.993, 1.016) | 1.006(0.987, 1.026) | 0.997(0.986, 1.007) | 0.997(0.963, 1.032) |  | 1.046(1.012, 1.080) ^b^ | 1.083(1.026, 1.144) ^b^ | 0.911(0.884, 0.939) ^b^ | 0.780(0.709, 0.858) ^b^ |
| 3 | 1.005(0.995, 1.014) | 1.006(0.991, 1.022) | 1.007(0.999, 1.016) | 1.034(1.006, 1.062) ^b^ |  | 1.053(1.016, 1.090) ^b^ | 1.095(1.033, 1.161) ^b^ | 0.920(0.891, 0.950) ^b^ | 0.816(0.737, 0.903) ^b^ |
| 4 | 1.007(0.998, 1.016) | 1.011(0.996, 1.027) | 1.010(1.002, 1.018) ^b^ | 1.046(1.018, 1.074) ^b^ |  | 1.060(1.020, 1.101) ^b^ | 1.109(1.041, 1.182) ^b^ | 0.929(0.898, 0.962) ^b^ | 0.853(0.764, 0.951) ^b^ |
| 5 | 1.007(0.996, 1.018) | 1.013(0.994, 1.032) | 1.010(1.000, 1.021) | 1.045(1.011, 1.081) ^b^ |  | 1.062(1.018, 1.107) ^b^ | 1.115(1.040, 1.195) ^b^ | 0.941(0.906, 0.977) ^b^ | 0.891(0.791, 1.003) |
| 6 | 1.002(0.992, 1.012) | 1.005(0.988, 1.022) | 1.013(1.004, 1.022) ^b^ | 1.045(1.015, 1.076) ^b^ |  | 1.048(1.002, 1.096) ^b^ | 1.092(1.015, 1.176) ^b^ | 0.963(0.926, 1.002) | 0.943(0.833, 1.068) |
| 7 | 0.987(0.969, 1.006) | 0.980(0.950, 1.011) | 1.023(1.005, 1.042) ^b^ | 1.058(0.999, 1.121) |  |  |  |  |  |
| O_3_ (μg/m^3^) |  |  |  |  |  | 0.994(0.986, 1.001) | 0.992(0.977, 1.007) | 1.012(1.001, 1.024) ^b^ | 1.027(1.001, 1.053) ^b^ |
| 0 | 0.994(0.986, 1.001) | 0.992(0.977, 1.007) | 1.012(1.001, 1.024) ^b^ | 1.027(1.001, 1.053) ^b^ |  | 0.988(0.977, 0.999) ^b^ | 0.985(0.962, 1.009) | 1.024(1.006, 1.043) ^b^ | 1.053(1.013, 1.095) ^b^ |
| 1 | 0.994(0.990, 0.999) ^b^ | 0.993(0.984, 1.002) | 1.012(1.005, 1.019) ^b^ | 1.025(1.010, 1.041) ^b^ |  | 0.983(0.969, 0.996) ^b^ | 0.979(0.952, 1.008) | 1.036(1.014, 1.058) ^b^ | 1.078(1.029, 1.129) ^b^ |
| 2 | 0.995(0.991, 0.998) ^b^ | 0.994(0.987, 1.002) | 1.011(1.005, 1.017) ^b^ | 1.024(1.011, 1.037) ^b^ |  | 0.978(0.964, 0.993) ^b^ | 0.975(0.945, 1.006) | 1.046(1.021, 1.071) ^b^ | 1.101(1.045, 1.159) ^b^ |
| 3 | 0.995(0.992, 0.999) ^b^ | 0.996(0.988, 1.003) | 1.010(1.003, 1.016) ^b^ | 1.021(1.007, 1.036) ^b^ |  | 0.975(0.959, 0.990) ^b^ | 0.972(0.940, 1.006) | 1.055(1.027, 1.083) ^b^ | 1.121(1.058, 1.187) ^b^ |
| 4 | 0.996(0.993, 1.000) ^b^ | 0.997(0.989, 1.005) | 1.009(1.002, 1.015) ^b^ | 1.018(1.004, 1.033) ^b^ |  | 0.972(0.955, 0.989) ^b^ | 0.970(0.935, 1.006) | 1.062(1.032, 1.094) ^b^ | 1.137(1.068, 1.211) ^b^ |
| 5 | 0.997(0.994, 1.000) | 0.998(0.991, 1.005) | 1.007(1.001, 1.013) ^b^ | 1.015(1.002, 1.027) ^b^ |  | 0.970(0.953, 0.988) ^b^ | 0.969(0.933, 1.007) | 1.068(1.036, 1.102) ^b^ | 1.149(1.074, 1.230) ^b^ |
| 6 | 0.998(0.994, 1.002) | 0.999(0.991, 1.007) | 1.006(0.999, 1.012) | 1.011(0.997, 1.025) |  | 0.969(0.950, 0.989) ^b^ | 0.970(0.930, 1.012) | 1.072(1.036, 1.110) ^b^ | 1.156(1.073, 1.247) ^b^ |
| 7 | 0.999(0.993, 1.006) | 1.001(0.987, 1.014) | 1.004(0.993, 1.015) | 1.006(0.982, 1.031) |  |  |  |  |  |
| PM_2.5_ (μg/m^3^) |  |  |  |  |  | 0.996(0.992, 1.001) | 0.992(0.985, 1.001) | 1.004(0.998, 1.009) | 1.007(0.995, 1.019) |
| 0 | 0.996(0.992, 1.001) | 0.992(0.985, 1.001) | 1.004(0.998, 1.009) | 1.007(0.995, 1.019) |  | 0.993(0.985, 1.001) | 0.986(0.972, 1.000) | 1.006(0.997, 1.016) | 1.012(0.992, 1.033) |
| 1 | 0.997(0.993, 1.000) | 0.993(0.987, 1.000) ^b^ | 1.003(0.998, 1.007) | 1.005(0.996, 1.015) |  | 0.991(0.980, 1.001) | 0.981(0.962, 1.000) ^b^ | 1.008(0.995, 1.022) | 1.016(0.988, 1.045) |
| 2 | 0.997(0.995, 1.000) | 0.994(0.989, 1.000) ^b^ | 1.002(0.999, 1.006) | 1.004(0.997, 1.011) |  | 0.989(0.976, 1.001) | 0.976(0.954, 0.999) ^b^ | 1.010(0.994, 1.025) | 1.019(0.986, 1.053) |
| 3 | 0.998(0.996, 1.000) | 0.995(0.991, 1.000) ^b^ | 1.001(0.999, 1.004) | 1.002(0.996, 1.008) |  | 0.987(0.974, 1.001) | 0.973(0.948, 0.998) ^b^ | 1.011(0.993, 1.028) | 1.019(0.983, 1.057) |
| 4 | 0.999(0.996, 1.001) | 0.996(0.993, 1.000) | 1.001(0.998, 1.003) | 1.001(0.995, 1.007) |  | 0.987(0.972, 1.002) | 0.970(0.944, 0.997) ^b^ | 1.011(0.992, 1.029) | 1.019(0.979, 1.060) |
| 5 | 0.999(0.997, 1.002) | 0.997(0.993, 1.002) | 1.000(0.997, 1.003) | 0.999(0.993, 1.006) |  | 0.986(0.970, 1.002) | 0.969(0.941, 0.997) ^b^ | 1.010(0.990, 1.030) | 1.017(0.974, 1.060) |
| 6 | 1.000(0.996, 1.003) | 0.998(0.993, 1.004) | 0.999(0.995, 1.003) | 0.998(0.989, 1.006) |  | 0.987(0.969, 1.004) | 0.968(0.938, 0.999) ^b^ | 1.008(0.987, 1.030) | 1.013(0.967, 1.061) |
| 7 | 1.000(0.996, 1.004) | 0.999(0.992, 1.007) | 0.999(0.994, 1.004) | 0.996(0.986, 1.007) |  | 1.023(0.931, 1.125) | 1.109(0.852, 1.442) | 0.953(0.873, 1.039) | 0.804(0.679, 0.953) ^b^ |

^a^: The median concentration of SO_2_ is 9 μg/m^3^, so defined 0 μg/m^3^ as the concentration reduced by 10 μg/m^3^, there is no the concentration reduced by 20 μg/m^3^.

^b^: *P*<0.05.

**Table S4**  The single-day and cumulative effects of air pollutants on female outpatients with psoriasis when changed by 10 or 20 μg/m^3^.

| Lag (d) | Single-day effects | | | |  | Cumulative effects | | | |
| --- | --- | --- | --- | --- | --- | --- | --- | --- | --- |
|  | 10 units increment | 20 units increment | 10 units decrement | 20 units decrement |  | 10 units increment | 20 units increment | 10 units decrement | 20 units decrement |
| SO_2_ (μg/m^3^) ^a^ |  |  |  |  |  |  |  |  |  |
| 0 | 1.032(0.997, 1.067) | 0.991(0.940, 1.043) | 1.027(0.961, 1.098) | - |  | 1.032(0.997, 1.067) | 0.991(0.940, 1.043) | 1.027(0.961, 1.098) | - |
| 1 | 1.027(1.000, 1.055) | 0.986(0.947, 1.028) | 1.028(0.975, 1.083) | - |  | 1.059(0.997, 1.125) | 0.977(0.890, 1.072) | 1.055(0.937, 1.189) | - |
| 2 | 1.022(1.001, 1.043) ^b^ | 0.982(0.952, 1.014) | 1.028(0.987, 1.071) | - |  | 1.083(0.999, 1.173) | 0.960(0.848, 1.086) | 1.085(0.926, 1.272) | - |
| 3 | 1.017(1.001, 1.034) ^b^ | 0.978(0.954, 1.003) | 1.029(0.996, 1.063) | - |  | 1.101(1.002, 1.210) ^b^ | 0.939(0.812, 1.085) | 1.117(0.927, 1.345) | - |
| 4 | 1.013(0.997, 1.029) | 0.974(0.951, 0.998) | 1.030(0.999, 1.062) | - |  | 1.115(1.006, 1.237) ^b^ | 0.915(0.780, 1.072) | 1.150(0.938, 1.410) | - |
| 5 | 1.008(0.989, 1.027) | 0.970(0.942, 0.999) | 1.031(0.993, 1.070) | - |  | 1.124(1.008, 1.254) ^b^ | 0.887(0.750, 1.049) | 1.186(0.956, 1.470) | - |
| 6 | 1.003(0.979, 1.028) | 0.966(0.930, 1.003) | 1.031(0.983, 1.082) | - |  | 1.128(1.005, 1.265) ^b^ | 0.857(0.718, 1.023) | 1.223(0.975, 1.533) | - |
| 7 | 0.999(0.967, 1.031) | 0.962(0.917, 1.009) | 1.032(0.970, 1.097) | - |  | 1.126(0.994, 1.276) | 0.825(0.681, 0.998) | 1.262(0.988, 1.611) | - |
| NO_2_ (μg/m^3^) |  |  |  |  |  |  |  |  |  |
| 0 | 1.029(1.010, 1.048) ^b^ | 1.046(1.014, 1.079) ^b^ | 0.932(0.915, 0.950) ^b^ | 0.826(0.779, 0.876) ^b^ |  | 1.029(1.010, 1.048) ^b^ | 1.046(1.014, 1.079) ^b^ | 0.932(0.915, 0.950) ^b^ | 0.826(0.779, 0.876) ^b^ |
| 1 | 1.022(1.011, 1.032) ^b^ | 1.036(1.018, 1.054) ^b^ | 0.963(0.953, 0.974) ^b^ | 0.912(0.883, 0.942) ^b^ |  | 1.051(1.022, 1.081) ^b^ | 1.084(1.034, 1.136) ^b^ | 0.898(0.872, 0.924) ^b^ | 0.753(0.689, 0.824) ^b^ |
| 2 | 1.015(1.007, 1.024) ^b^ | 1.027(1.012, 1.041) ^b^ | 0.988(0.981, 0.995) ^b^ | 0.983(0.958, 1.007) |  | 1.067(1.033, 1.102) ^b^ | 1.112(1.054, 1.174) ^b^ | 0.887(0.858, 0.917) ^b^ | 0.740(0.668, 0.820) ^b^ |
| 3 | 1.010(1.000, 1.019) ^b^ | 1.018(1.002, 1.034) ^b^ | 1.006(0.997, 1.014) | 1.033(1.005, 1.062) ^b^ |  | 1.078(1.040, 1.116) ^b^ | 1.133(1.068, 1.201) ^b^ | 0.892(0.862, 0.924) ^b^ | 0.765(0.686, 0.853) ^b^ |
| 4 | 1.005(0.996, 1.015) | 1.011(0.995, 1.027) | 1.016(1.007, 1.024) ^b^ | 1.061(1.032, 1.091) ^b^ |  | 1.083(1.042, 1.125) ^b^ | 1.145(1.074, 1.220) ^b^ | 0.906(0.874, 0.940) ^b^ | 0.812(0.723, 0.911) ^b^ |
| 5 | 1.001(0.993, 1.009) | 1.004(0.991, 1.018) | 1.018(1.011, 1.025) ^b^ | 1.064(1.038, 1.089) ^b^ |  | 1.084(1.040, 1.130) ^b^ | 1.149(1.073, 1.231) ^b^ | 0.922(0.888, 0.958) ^b^ | 0.863(0.765, 0.974) ^b^ |
| 6 | 0.998(0.989, 1.008) | 0.998(0.983, 1.014) | 1.013(1.005, 1.021) ^b^ | 1.041(1.012, 1.070) ^b^ |  | 1.083(1.036, 1.132) ^b^ | 1.148(1.067, 1.234) ^b^ | 0.934(0.899, 0.971) ^b^ | 0.898(0.792, 1.019) |
| 7 | 0.996(0.980, 1.013) | 0.994(0.966, 1.022) | 1.000(0.985, 1.015) | 0.994(0.945, 1.046) |  | 1.079(1.026, 1.134) ^b^ | 1.140(1.051, 1.237) ^b^ | 0.934(0.894, 0.976) ^b^ | 0.893(0.776, 1.026) |
| O_3_ (μg/m^3^) |  |  |  |  |  |  |  |  |  |
| 0 | 1.000(0.994, 1.006) | 1.001(0.989, 1.013) | 1.002(0.996, 1.008) | 1.007(0.997, 1.018) |  | 1.000(0.994, 1.006) | 1.001(0.989, 1.013) | 1.002(0.996, 1.008) | 1.007(0.997, 1.018) |
| 1 | 1.000(0.995, 1.005) | 1.001(0.991, 1.011) | 1.001(0.997, 1.006) | 1.005(0.997, 1.014) |  | 1.000(0.989, 1.012) | 1.002(0.980, 1.024) | 1.003(0.993, 1.013) | 1.013(0.994, 1.032) |
| 2 | 1.000(0.996, 1.004) | 1.000(0.993, 1.008) | 1.001(0.997, 1.004) | 1.003(0.997, 1.010) |  | 1.000(0.986, 1.015) | 1.002(0.973, 1.032) | 1.004(0.991, 1.018) | 1.016(0.991, 1.042) |
| 3 | 1.000(0.997, 1.003) | 1.000(0.994, 1.006) | 1.000(0.998, 1.003) | 1.001(0.996, 1.006) |  | 1.000(0.983, 1.018) | 1.002(0.968, 1.037) | 1.005(0.989, 1.021) | 1.017(0.988, 1.048) |
| 4 | 1.000(0.997, 1.003) | 0.999(0.994, 1.005) | 1.000(0.997, 1.002) | 0.999(0.994, 1.004) |  | 1.000(0.981, 1.020) | 1.002(0.964, 1.040) | 1.004(0.987, 1.022) | 1.016(0.984, 1.050) |
| 5 | 1.000(0.996, 1.003) | 0.999(0.992, 1.006) | 0.999(0.996, 1.002) | 0.997(0.992, 1.003) |  | 1.000(0.979, 1.021) | 1.001(0.961, 1.042) | 1.004(0.985, 1.022) | 1.013(0.979, 1.049) |
| 6 | 1.000(0.995, 1.004) | 0.999(0.990, 1.007) | 0.999(0.995, 1.003) | 0.995(0.988, 1.002) |  | 0.999(0.978, 1.021) | 0.999(0.957, 1.043) | 1.002(0.983, 1.022) | 1.008(0.973, 1.045) |
| 7 | 0.999(0.994, 1.005) | 0.998(0.987, 1.009) | 0.998(0.993, 1.003) | 0.993(0.984, 1.002) |  | 0.999(0.976, 1.023) | 0.997(0.952, 1.045) | 1.001(0.980, 1.022) | 1.001(0.964, 1.040) |
| PM_2.5_ (μg/m^3^) |  |  |  |  |  |  |  |  |  |
| 0 | 1.004(0.997, 1.011) | 1.007(0.994, 1.021) | 1.003(0.997, 1.009) | 1.019(1.002, 1.035) ^b^ |  | 1.004(0.997, 1.011) | 1.007(0.994, 1.021) | 1.003(0.997, 1.009) | 1.019(1.002, 1.035) |
| 1 | 1.003(0.998, 1.009) | 1.006(0.995, 1.018) | 1.002(0.998, 1.007) | 1.015(1.002, 1.028) ^b^ |  | 1.007(0.994, 1.020) | 1.014(0.989, 1.039) | 1.006(0.995, 1.016) | 1.034(1.004, 1.064) |
| 2 | 1.003(0.998, 1.008) | 1.006(0.997, 1.014) | 1.001(0.998, 1.005) | 1.011(1.001, 1.021) ^b^ |  | 1.010(0.993, 1.027) | 1.019(0.986, 1.054) | 1.007(0.993, 1.022) | 1.045(1.005, 1.087) |
| 3 | 1.003(0.999, 1.006) | 1.005(0.997, 1.012) | 1.001(0.997, 1.004) | 1.007(0.999, 1.016) |  | 1.012(0.992, 1.033) | 1.024(0.984, 1.065) | 1.008(0.991, 1.025) | 1.053(1.005, 1.103) |
| 4 | 1.002(0.998, 1.006) | 1.004(0.996, 1.011) | 1.000(0.997, 1.003) | 1.003(0.995, 1.012) |  | 1.015(0.992, 1.038) | 1.028(0.983, 1.075) | 1.007(0.988, 1.027) | 1.056(1.003, 1.112) |
| 5 | 1.002(0.997, 1.006) | 1.003(0.994, 1.012) | 0.999(0.995, 1.002) | 1.000(0.990, 1.009) |  | 1.017(0.991, 1.042) | 1.031(0.982, 1.082) | 1.006(0.986, 1.027) | 1.056(0.999, 1.116) |
| 6 | 1.002(0.996, 1.007) | 1.002(0.991, 1.013) | 0.998(0.994, 1.002) | 0.996(0.984, 1.008) |  | 1.018(0.991, 1.046) | 1.033(0.980, 1.089) | 1.004(0.982, 1.026) | 1.051(0.991, 1.115) |
| 7 | 1.001(0.994, 1.008) | 1.001(0.988, 1.014) | 0.997(0.992, 1.003) | 0.992(0.977, 1.007) |  | 1.019(0.989, 1.051) | 1.034(0.976, 1.096) | 1.001(0.977, 1.025) | 1.043(0.978, 1.112) |

^a^: The median concentration of SO_2_ is 9 μg/m^3^, so defined 0 μg/m^3^ as the concentration reduced by 10 μg/m^3^, there is no the concentration reduced by 20 μg/m^3^.

^b^: *P*<0.05.

**Table S5**  The single-day and cumulative effects of air pollutants on outpatients with psoriasis who aged 0-17 years old when changed by 10 or 20 μg/m^3^.

| Lag (d) | Single-day effects | | | |  | Cumulative effects | | | |
| --- | --- | --- | --- | --- | --- | --- | --- | --- | --- |
|  | 10 units increment | 20 units increment | 10 units decrement | 20 units decrement |  | 10 units increment | 20 units increment | 10 units decrement | 20 units decrement |
| SO_2_ (μg/m^3^) ^a^ |  |  |  |  |  |  |  |  |  |
| 0 | 0.961(0.908, 1.017) | 0.858(0.768, 0.959) ^b^ | 0.973(0.888, 1.066) | - |  | 0.961(0.908, 1.017) | 0.858(0.768, 0.959) ^b^ | 0.973(0.888, 1.066) | - |
| 1 | 0.972(0.930, 1.017) | 0.877(0.803, 0.958) ^b^ | 0.962(0.896, 1.034) | - |  | 0.934(0.845, 1.034) | 0.752(0.616, 0.919) ^b^ | 0.937(0.796, 1.102) | - |
| 2 | 0.983(0.950, 1.017) | 0.896(0.837, 0.960) ^b^ | 0.952(0.901, 1.005) | - |  | 0.919(0.804, 1.050) | 0.674(0.517, 0.880) ^b^ | 0.892(0.719, 1.106) | - |
| 3 | 0.994(0.969, 1.020) | 0.916(0.868, 0.967) ^b^ | 0.942(0.903, 0.982) ^b^ | - |  | 0.914(0.781, 1.068) | 0.617(0.452, 0.844) ^b^ | 0.840(0.653, 1.080) | - |
| 4 | 1.006(0.983, 1.029) | 0.936(0.889, 0.984) ^b^ | 0.931(0.896, 0.967) ^b^ | - |  | 0.919(0.776, 1.088) | 0.578(0.410, 0.813) ^b^ | 0.782(0.595, 1.028) | - |
| 5 | 1.017(0.990, 1.046) | 0.956(0.900, 1.016) | 0.921(0.880, 0.964) ^b^ | - |  | 0.935(0.784, 1.114) | 0.552(0.385, 0.793) ^b^ | 0.720(0.542, 0.957) ^b^ | - |
| 6 | 1.029(0.992, 1.067) | 0.977(0.904, 1.057) | 0.911(0.857, 0.968) ^b^ | - |  | 0.961(0.803, 1.151) | 0.540(0.370, 0.788) ^b^ | 0.656(0.489, 0.879) ^b^ | - |
| 7 | 1.040(0.992, 1.091) | 0.999(0.903, 1.104) | 0.901(0.833, 0.975) ^b^ | - |  | 1.000(0.829, 1.207) | 0.539(0.359, 0.809) ^b^ | 0.591(0.434, 0.805) ^b^ | - |
| NO_2_ (μg/m^3^) |  |  |  |  |  |  |  |  |  |
| 0 | 1.044(1.005, 1.084) ^b^ | 1.111(1.026, 1.204) ^b^ | 0.961(0.896, 1.031) | 0.699(0.621, 0.786) ^b^ |  | 1.044(1.005, 1.084) ^b^ | 1.111(1.026, 1.204) ^b^ | 0.961(0.896, 1.031) | 0.699(0.621, 0.786) ^b^ |
| 1 | 1.027(1.006, 1.049) ^b^ | 1.069(1.021, 1.119) ^b^ | 0.991(0.951, 1.033) | 0.862(0.808, 0.919) ^b^ |  | 1.073(1.013, 1.136) ^b^ | 1.188(1.052, 1.342) ^b^ | 0.952(0.855, 1.061) | 0.602(0.504, 0.719) ^b^ |
| 2 | 1.016(0.999, 1.033) | 1.040(1.002, 1.080) ^b^ | 1.015(0.979, 1.053) | 1.008(0.959, 1.060) |  | 1.090(1.020, 1.164) ^b^ | 1.236(1.072, 1.425) ^b^ | 0.967(0.850, 1.100) | 0.607(0.495, 0.744) ^b^ |
| 3 | 1.009(0.991, 1.029) | 1.025(0.983, 1.069) | 1.033(0.992, 1.075) | 1.118(1.057, 1.183) |  | 1.100(1.024, 1.182) ^b^ | 1.267(1.084, 1.481) ^b^ | 0.998(0.864, 1.154) | 0.679(0.547, 0.843) ^b^ |
| 4 | 1.008(0.989, 1.027) | 1.021(0.979, 1.066) | 1.043(1.002, 1.085) | 1.175(1.111, 1.244) ^b^ |  | 1.108(1.026, 1.198) ^b^ | 1.294(1.091, 1.535) ^b^ | 1.041(0.885, 1.225) | 0.798(0.634, 1.004) |
| 5 | 1.010(0.994, 1.027) | 1.030(0.993, 1.069) | 1.046(1.009, 1.084) | 1.172(1.116, 1.230) ^b^ |  | 1.120(1.030, 1.217) ^b^ | 1.333(1.107, 1.606) ^b^ | 1.089(0.911, 1.302) | 0.935(0.732, 1.193) |
| 6 | 1.018(0.999, 1.037) | 1.052(1.009, 1.096) | 1.041(1.000, 1.084) | 1.107(1.046, 1.172) ^b^ |  | 1.140(1.043, 1.246) ^b^ | 1.402(1.150, 1.711) ^b^ | 1.133(0.935, 1.373) | 1.035(0.801, 1.337) |
| 7 | 1.030(0.996, 1.066) | 1.087(1.010, 1.169) | 1.029(0.961, 1.102) | 0.992(0.893, 1.102) |  | 1.175(1.064, 1.298) ^b^ | 1.524(1.221, 1.902) ^b^ | 1.166(0.943, 1.443) | 1.027(0.770, 1.370) |
| O_3_ (μg/m^3^) |  |  |  |  |  |  |  |  |  |
| 0 | 1.019(1.000, 1.038) ^b^ | 1.028(0.989, 1.069) | 0.973(0.946, 1.001) | 0.948(0.892, 1.007) |  | 1.019(1.000, 1.038) ^b^ | 1.028(0.989, 1.069) | 0.973(0.946, 1.001) | 0.948(0.892, 1.007) |
| 1 | 1.003(0.992, 1.014) | 1.010(0.988, 1.033) | 1.003(0.987, 1.020) | 1.009(0.974, 1.046) |  | 1.022(0.994, 1.052) | 1.039(0.979, 1.102) | 0.976(0.935, 1.019) | 0.957(0.872, 1.050) |
| 2 | 0.993(0.984, 1.001) | 0.998(0.979, 1.017) | 1.023(1.009, 1.038) ^b^ | 1.050(1.018, 1.083) ^b^ |  | 1.015(0.982, 1.049) | 1.036(0.966, 1.112) | 0.999(0.949, 1.051) | 1.005(0.899, 1.123) |
| 3 | 0.988(0.978, 0.997) ^b^ | 0.991(0.971, 1.011) | 1.032(1.016, 1.048) ^b^ | 1.067(1.031, 1.103) ^b^ |  | 1.002(0.966, 1.039) | 1.027(0.950, 1.110) | 1.030(0.973, 1.091) | 1.071(0.946, 1.214) |
| 4 | 0.988(0.979, 0.997) ^b^ | 0.989(0.970, 1.010) | 1.028(1.013, 1.045) ^b^ | 1.058(1.023, 1.095) ^b^ |  | 0.990(0.951, 1.030) | 1.016(0.933, 1.107) | 1.060(0.994, 1.129) | 1.134(0.986, 1.304) |
| 5 | 0.993(0.985, 1.001) | 0.993(0.976, 1.011) | 1.014(1.000, 1.028) ^b^ | 1.026(0.995, 1.058) |  | 0.983(0.942, 1.026) | 1.009(0.920, 1.107) | 1.075(1.002, 1.153) ^b^ | 1.163(0.998, 1.357) |
| 6 | 1.004(0.995, 1.013) | 1.003(0.983, 1.023) | 0.989(0.973, 1.005) | 0.971(0.938, 1.006) |  | 0.987(0.944, 1.032) | 1.012(0.918, 1.116) | 1.062(0.985, 1.146) | 1.130(0.957, 1.334) |
| 7 | 1.020(1.004, 1.037) ^b^ | 1.017(0.983, 1.053) | 0.953(0.928, 0.979) ^b^ | 0.898(0.847, 0.953) ^b^ |  | 1.007(0.958, 1.058) | 1.029(0.925, 1.146) | 1.013(0.930, 1.102) | 1.015(0.843, 1.222) |
| PM_2.5_ (μg/m^3^) |  |  |  |  |  |  |  |  |  |
| 0 | 0.984(0.973, 0.995) ^b^ | 0.968(0.949, 0.988) ^b^ | 1.017(1.004, 1.031) ^b^ | 1.035(1.006, 1.065) ^b^ |  | 0.984(0.973, 0.995) ^b^ | 0.968(0.949, 0.988) ^b^ | 1.017(1.004, 1.031) ^b^ | 1.035(1.006, 1.065) ^b^ |
| 1 | 0.987(0.979, 0.996) ^b^ | 0.975(0.960, 0.991) ^b^ | 1.013(1.002, 1.024) ^b^ | 1.026(1.003, 1.050) ^b^ |  | 0.971(0.952, 0.991) ^b^ | 0.944(0.911, 0.979) ^b^ | 1.030(1.006, 1.055) ^b^ | 1.062(1.009, 1.118) ^b^ |
| 2 | 0.991(0.984, 0.998) ^b^ | 0.982(0.969, 0.994) ^b^ | 1.009(1.000, 1.017) ^b^ | 1.017(0.999, 1.036) |  | 0.963(0.937, 0.989) ^b^ | 0.927(0.883, 0.973) ^b^ | 1.039(1.006, 1.073) ^b^ | 1.081(1.009, 1.157) ^b^ |
| 3 | 0.995(0.989, 1.000) | 0.988(0.978, 0.999) ^b^ | 1.004(0.997, 1.011) | 1.008(0.993, 1.023) |  | 0.957(0.928, 0.988) ^b^ | 0.916(0.865, 0.970) ^b^ | 1.044(1.005, 1.084) ^b^ | 1.089(1.004, 1.182) ^b^ |
| 4 | 0.998(0.993, 1.004) | 0.995(0.985, 1.005) | 1.000(0.994, 1.007) | 0.999(0.985, 1.014) |  | 0.956(0.923, 0.990) ^b^ | 0.912(0.856, 0.971) ^b^ | 1.044(1.001, 1.089) ^b^ | 1.089(0.994, 1.192) |
| 5 | 1.002(0.996, 1.008) | 1.002(0.990, 1.013) | 0.996(0.988, 1.004) | 0.990(0.974, 1.007) |  | 0.958(0.922, 0.994) ^b^ | 0.914(0.854, 0.978) ^b^ | 1.040(0.994, 1.089) | 1.078(0.978, 1.189) |
| 6 | 1.006(0.998, 1.014) | 1.009(0.994, 1.023) | 0.992(0.982, 1.002) | 0.982(0.961, 1.003) |  | 0.963(0.925, 1.003) | 0.921(0.857, 0.991) ^b^ | 1.031(0.982, 1.083) | 1.058(0.953, 1.176) |
| 7 | 1.009(0.999, 1.020) | 1.016(0.997, 1.034) | 0.988(0.976, 1.000) ^b^ | 0.973(0.948, 0.999) ^b^ |  | 0.972(0.931, 1.015) | 0.936(0.865, 1.012) | 1.019(0.966, 1.075) | 1.030(0.918, 1.155) |

^a^: The median concentration of SO_2_ is 9 μg/m^3^, so defined 0 μg/m^3^ as the concentration reduced by 10 μg/m^3^, there is no the concentration reduced by 20 μg/m^3^.

^b^: *P*<0.05.

**Table S6**  The single-day and cumulative effects of air pollutants on outpatients with psoriasis who aged 18-39 years old when changed by 10 or 20 μg/m^3^.

| Lag (d) | Single-day effects | | | |  | Cumulative effects | | | |
| --- | --- | --- | --- | --- | --- | --- | --- | --- | --- |
|  | 10 units increment | 20 units increment | 10 units decrement | 20 units decrement |  | 10 units increment | 20 units increment | 10 units decrement | 20 units decrement |
| SO_2_ (μg/m^3^) ^a^ |  |  |  |  |  |  |  |  |  |
| 0 | 0.986(0.939, 1.036) | 0.919(0.851, 0.992) ^b^ | 1.097(0.990, 1.215) | - |  | 0.986(0.939, 1.036) | 0.919(0.851, 0.992) ^b^ | 1.097(0.990, 1.215) | - |
| 1 | 1.005(0.978, 1.032) | 0.960(0.921, 1.002) | 1.053(0.997, 1.112) | - |  | 0.991(0.920, 1.067) | 0.882(0.786, 0.990) ^b^ | 1.155(0.992, 1.344) | - |
| 2 | 1.016(0.995, 1.038) | 0.989(0.957, 1.022) | 1.024(0.981, 1.068) | - |  | 1.007(0.925, 1.096) | 0.873(0.764, 0.996) ^b^ | 1.182(0.995, 1.404) | - |
| 3 | 1.021(0.997, 1.047) | 1.003(0.966, 1.040) | 1.009(0.960, 1.060) | - |  | 1.029(0.939, 1.127) | 0.875(0.759, 1.008) | 1.193(0.994, 1.430) | - |
| 4 | 1.019(0.995, 1.045) | 1.001(0.964, 1.040) | 1.007(0.958, 1.059) | - |  | 1.049(0.951, 1.156) | 0.876(0.753, 1.018) | 1.201(0.990, 1.457) | - |
| 5 | 1.011(0.989, 1.033) | 0.985(0.954, 1.018) | 1.018(0.976, 1.062) | - |  | 1.060(0.956, 1.176) | 0.863(0.736, 1.012) | 1.223(0.996, 1.502) | - |
| 6 | 0.995(0.972, 1.020) | 0.955(0.920, 0.990) ^b^ | 1.044(0.995, 1.094) | - |  | 1.055(0.947, 1.176) | 0.824(0.697, 0.973) ^b^ | 1.277(1.030, 1.583) ^b^ | - |
| 7 | 0.974(0.932, 1.017) | 0.911(0.853, 0.974) ^b^ | 1.083(0.991, 1.185) | - |  | 1.027(0.911, 1.158) | 0.751(0.625, 0.902) ^b^ | 1.383(1.088, 1.759) ^b^ | - |
| NO_2_ (μg/m^3^) |  |  |  |  |  |  |  |  |  |
| 0 | 1.026(1.004, 1.048) ^b^ | 1.044(1.007, 1.083) ^b^ | 0.926(0.906, 0.947) ^b^ | 0.803(0.750, 0.859) ^b^ |  | 1.026(1.004, 1.048) ^b^ | 1.044(1.007, 1.083) ^b^ | 0.926(0.906, 0.947) ^b^ | 0.803(0.750, 0.859) ^b^ |
| 1 | 1.015(1.004, 1.026) ^b^ | 1.027(1.008, 1.046) ^b^ | 0.975(0.965, 0.985) ^b^ | 0.939(0.908, 0.971) ^b^ |  | 1.041(1.014, 1.069) ^b^ | 1.072(1.025, 1.122) ^b^ | 0.903(0.879, 0.928) ^b^ | 0.754(0.693, 0.820) ^b^ |
| 2 | 1.010(0.998, 1.022) | 1.018(0.997, 1.039) | 1.000(0.989, 1.012) | 1.016(0.980, 1.054) |  | 1.052(1.021, 1.083) ^b^ | 1.091(1.038, 1.147) ^b^ | 0.903(0.877, 0.930) ^b^ | 0.766(0.698, 0.840) ^b^ |
| 3 | 1.008(0.998, 1.018) | 1.013(0.997, 1.030) | 1.009(1.000, 1.017) | 1.042(1.012, 1.073) ^b^ |  | 1.060(1.025, 1.095) ^b^ | 1.106(1.046, 1.169) ^b^ | 0.911(0.883, 0.940) ^b^ | 0.798(0.722, 0.882) ^b^ |
| 4 | 1.007(0.997, 1.017) | 1.011(0.995, 1.028) | 1.007(0.998, 1.016) | 1.035(1.006, 1.065) ^b^ |  | 1.067(1.029, 1.106) ^b^ | 1.118(1.053, 1.187) ^b^ | 0.917(0.888, 0.948) ^b^ | 0.826(0.743, 0.918) ^b^ |
| 5 | 1.005(0.993, 1.017) | 1.008(0.988, 1.029) | 1.003(0.992, 1.014) | 1.019(0.983, 1.056) |  | 1.072(1.031, 1.115) ^b^ | 1.127(1.056, 1.203) ^b^ | 0.920(0.888, 0.954) ^b^ | 0.842(0.750, 0.944) ^b^ |
| 6 | 1.001(0.990, 1.011) | 1.001(0.984, 1.019) | 1.005(0.996, 1.015) | 1.018(0.987, 1.050) |  | 1.073(1.027, 1.120) ^b^ | 1.129(1.051, 1.213) ^b^ | 0.925(0.890, 0.962) ^b^ | 0.857(0.757, 0.971) ^b^ |
| 7 | 0.992(0.972, 1.012) | 0.988(0.956, 1.022) | 1.020(1.001, 1.040) ^b^ | 1.056(0.993, 1.122) |  | 1.064(1.016, 1.115) ^b^ | 1.115(1.034, 1.204) ^b^ | 0.944(0.907, 0.983) ^b^ | 0.905(0.795, 1.030) |
| O_3_ (μg/m^3^) |  |  |  |  |  |  |  |  |  |
| 0 | 0.993(0.988, 0.997) ^b^ | 0.986(0.977, 0.995) ^b^ | 1.007(1.003, 1.012) ^b^ | 1.015(1.005, 1.024) ^b^ |  | 0.993(0.988, 0.997) ^b^ | 0.986(0.977, 0.995) ^b^ | 1.007(1.003, 1.012) ^b^ | 1.015(1.005, 1.024) ^b^ |
| 1 | 0.996(0.993, 0.998) ^b^ | 0.991(0.986, 0.996) ^b^ | 1.004(1.002, 1.007) ^b^ | 1.009(1.004, 1.014) ^b^ |  | 0.988(0.982, 0.995) ^b^ | 0.977(0.963, 0.990) ^b^ | 1.012(1.005, 1.019) ^b^ | 1.024(1.010, 1.038) ^b^ |
| 2 | 0.998(0.996, 1.000) ^b^ | 0.996(0.992, 0.999) ^b^ | 1.002(1.000, 1.004) ^b^ | 1.004(1.001, 1.008) ^b^ |  | 0.986(0.978, 0.994) ^b^ | 0.973(0.957, 0.988) ^b^ | 1.014(1.006, 1.022) ^b^ | 1.028(1.012, 1.045) ^b^ |
| 3 | 0.999(0.997, 1.001) | 0.999(0.995, 1.003) | 1.001(0.999, 1.003) | 1.001(0.997, 1.005) |  | 0.986(0.977, 0.994) ^b^ | 0.971(0.955, 0.988) ^b^ | 1.015(1.006, 1.023) ^b^ | 1.030(1.013, 1.047) ^b^ |
| 4 | 1.000(0.998, 1.002) | 1.001(0.997, 1.005) | 1.000(0.998, 1.002) | 0.999(0.995, 1.003) |  | 0.986(0.977, 0.994) ^b^ | 0.972(0.955, 0.989) ^b^ | 1.014(1.006, 1.023) ^b^ | 1.029(1.011, 1.047) ^b^ |
| 5 | 1.001(0.999, 1.002) | 1.001(0.998, 1.005) | 0.999(0.998, 1.001) | 0.999(0.995, 1.002) |  | 0.987(0.978, 0.995) ^b^ | 0.973(0.956, 0.991) ^b^ | 1.014(1.005, 1.023) ^b^ | 1.028(1.009, 1.046) ^b^ |
| 6 | 1.000(0.998, 1.002) | 1.001(0.997, 1.005) | 1.000(0.998, 1.002) | 0.999(0.995, 1.003) |  | 0.987(0.978, 0.996) ^b^ | 0.974(0.956, 0.992) ^b^ | 1.013(1.004, 1.023) ^b^ | 1.027(1.008, 1.046) ^b^ |
| 7 | 0.999(0.996, 1.003) | 0.999(0.991, 1.006) | 1.001(0.997, 1.004) | 1.001(0.994, 1.009) |  | 0.986(0.976, 0.996) ^b^ | 0.973(0.953, 0.993) ^b^ | 1.014(1.004, 1.024) ^b^ | 1.028(1.007, 1.049) ^b^ |
| PM_2.5_ (μg/m^3^) |  |  |  |  |  |  |  |  |  |
| 0 | 0.998(0.994, 1.003) | 0.996(0.988, 1.005) | 1.001(0.996, 1.007) | 1.003(0.990, 1.015) |  | 0.998(0.994, 1.003) | 0.996(0.988, 1.005) | 1.001(0.996, 1.007) | 1.003(0.990, 1.015) |
| 1 | 0.999(0.995, 1.002) | 0.997(0.990, 1.004) | 1.001(0.996, 1.006) | 1.002(0.992, 1.011) |  | 0.997(0.989, 1.006) | 0.993(0.978, 1.009) | 1.002(0.992, 1.013) | 1.004(0.982, 1.026) |
| 2 | 0.999(0.996, 1.002) | 0.998(0.992, 1.003) | 1.000(0.997, 1.004) | 1.000(0.993, 1.008) |  | 0.996(0.985, 1.007) | 0.991(0.971, 1.012) | 1.003(0.989, 1.017) | 1.005(0.975, 1.034) |
| 3 | 0.999(0.997, 1.002) | 0.998(0.994, 1.003) | 1.000(0.997, 1.003) | 0.999(0.993, 1.006) |  | 0.995(0.982, 1.009) | 0.989(0.965, 1.013) | 1.003(0.986, 1.019) | 1.004(0.969, 1.040) |
| 4 | 1.000(0.997, 1.002) | 0.999(0.995, 1.003) | 0.999(0.997, 1.002) | 0.998(0.992, 1.004) |  | 0.995(0.980, 1.010) | 0.988(0.961, 1.015) | 1.002(0.984, 1.020) | 1.002(0.964, 1.042) |
| 5 | 1.000(0.997, 1.003) | 0.999(0.994, 1.004) | 0.999(0.996, 1.002) | 0.997(0.990, 1.004) |  | 0.995(0.979, 1.011) | 0.987(0.959, 1.016) | 1.001(0.982, 1.021) | 0.999(0.958, 1.042) |
| 6 | 1.000(0.997, 1.004) | 1.000(0.994, 1.006) | 0.998(0.994, 1.003) | 0.996(0.987, 1.005) |  | 0.996(0.979, 1.013) | 0.987(0.957, 1.018) | 1.000(0.979, 1.021) | 0.995(0.952, 1.041) |
| 7 | 1.001(0.997, 1.005) | 1.000(0.993, 1.008) | 0.998(0.993, 1.003) | 0.995(0.984, 1.006) |  | 0.997(0.978, 1.015) | 0.987(0.955, 1.021) | 0.998(0.975, 1.021) | 0.990(0.943, 1.040) |

^a^: The median concentration of SO_2_ is 9 μg/m^3^, so defined 0 μg/m^3^ as the concentration reduced by 10 μg/m^3^, there is no the concentration reduced by 20 μg/m^3^.

^b^: *P*<0.05.

**Table S7**  The single-day and cumulative effects of air pollutants on outpatients with psoriasis who aged 40-64 years old when changed by 10 or 20 μg/m^3^.

| Lag (d) | Single-day effects | | | |  | Cumulative effects | | | |
| --- | --- | --- | --- | --- | --- | --- | --- | --- | --- |
|  | 10 units increment | 20 units increment | 10 units decrement | 20 units decrement |  | 10 units increment | 20 units increment | 10 units decrement | 20 units decrement |
| SO_2_ (μg/m^3^) ^a^ |  |  |  |  |  |  |  |  |  |
| 0 | 1.005(0.979, 1.032) | 0.985(0.934, 1.040) | 0.974(0.933, 1.017) | - |  | 1.005(0.979, 1.032) | 0.985(0.934, 1.040) | 0.974(0.933, 1.017) | - |
| 1 | 1.001(0.980, 1.022) | 0.979(0.938, 1.021) | 0.979(0.946, 1.013) | - |  | 1.007(0.960, 1.055) | 0.964(0.876, 1.061) | 0.953(0.882, 1.030) | - |
| 2 | 0.997(0.981, 1.013) | 0.972(0.941, 1.005) | 0.983(0.958, 1.010) | - |  | 1.004(0.943, 1.069) | 0.937(0.825, 1.065) | 0.937(0.846, 1.039) | - |
| 3 | 0.993(0.981, 1.005) | 0.966(0.941, 0.991) ^b^ | 0.988(0.968, 1.008) | - |  | 0.997(0.926, 1.073) | 0.905(0.779, 1.052) | 0.926(0.822, 1.045) | - |
| 4 | 0.989(0.978, 1.000) ^b^ | 0.959(0.936, 0.983) ^b^ | 0.993(0.975, 1.011) | - |  | 0.986(0.910, 1.068) | 0.868(0.736, 1.024) | 0.920(0.807, 1.049) | - |
| 5 | 0.985(0.972, 0.998) ^b^ | 0.953(0.925, 0.982) ^b^ | 0.998(0.977, 1.020) | - |  | 0.971(0.894, 1.055) | 0.827(0.694, 0.985) ^b^ | 0.918(0.801, 1.053) | - |
| 6 | 0.981(0.964, 0.998) ^b^ | 0.946(0.911, 0.984) ^b^ | 1.003(0.975, 1.032) | - |  | 0.953(0.875, 1.037) | 0.783(0.651, 0.941) ^b^ | 0.921(0.799, 1.061) | - |
| 7 | 0.977(0.955, 0.999) ^b^ | 0.940(0.895, 0.987) ^b^ | 1.008(0.971, 1.045) | - |  | 0.931(0.852, 1.017) | 0.736(0.603, 0.897) ^b^ | 0.928(0.799, 1.077) | - |
| NO_2_ (μg/m^3^) |  |  |  |  |  |  |  |  |  |
| 0 | 1.013(0.994, 1.032) | 1.025(0.992, 1.059) | 0.956(0.938, 0.974) ^b^ | 0.874(0.823, 0.928) ^b^ |  | 1.013(0.994, 1.032) | 1.025(0.992, 1.059) | 0.956(0.938, 0.974) ^b^ | 0.874(0.823, 0.928) ^b^ |
| 1 | 1.009(0.998, 1.020) | 1.018(0.999, 1.037) | 0.975(0.965, 0.986) ^b^ | 0.931(0.900, 0.963) ^b^ |  | 1.022(0.993, 1.053) | 1.043(0.993, 1.096) | 0.932(0.905, 0.960) ^b^ | 0.814(0.742, 0.892) ^b^ |
| 2 | 1.006(0.996, 1.015) | 1.011(0.996, 1.026) | 0.991(0.983, 0.999) ^b^ | 0.978(0.952, 1.005) |  | 1.028(0.993, 1.064) | 1.055(0.995, 1.118) | 0.924(0.893, 0.956) ^b^ | 0.796(0.715, 0.886) ^b^ |
| 3 | 1.002(0.992, 1.012) | 1.005(0.988, 1.022) | 1.003(0.995, 1.012) | 1.014(0.984, 1.044) |  | 1.030(0.991, 1.070) | 1.059(0.994, 1.129) | 0.927(0.894, 0.962) ^b^ | 0.807(0.718, 0.906) ^b^ |
| 4 | 0.998(0.988, 1.008) | 0.998(0.982, 1.015) | 1.012(1.003, 1.020) ^b^ | 1.036(1.006, 1.067) ^b^ |  | 1.028(0.986, 1.072) | 1.058(0.986, 1.134) | 0.938(0.902, 0.975) ^b^ | 0.836(0.737, 0.947) ^b^ |
| 5 | 0.995(0.986, 1.003) | 0.993(0.978, 1.007) | 1.016(1.008, 1.023) ^b^ | 1.045(1.018, 1.072) ^b^ |  | 1.023(0.976, 1.071) | 1.050(0.973, 1.133) | 0.953(0.914, 0.993) ^b^ | 0.873(0.763, 0.999) ^b^ |
| 6 | 0.991(0.981, 1.001) | 0.987(0.971, 1.004) | 1.016(1.007, 1.025) ^b^ | 1.039(1.008, 1.070) ^b^ |  | 1.014(0.964, 1.065) | 1.037(0.955, 1.125) | 0.968(0.926, 1.011) | 0.907(0.786, 1.046) |
| 7 | 0.988(0.970, 1.006) | 0.982(0.953, 1.012) | 1.012(0.996, 1.028) | 1.019(0.965, 1.075) |  | 1.001(0.946, 1.059) | 1.018(0.929, 1.116) | 0.979(0.932, 1.029) | 0.924(0.787, 1.084) |
| O_3_ (μg/m^3^) |  |  |  |  |  |  |  |  |  |
| 0 | 0.992(0.987, 0.998) ^b^ | 0.985(0.974, 0.996) ^b^ | 1.008(1.002, 1.013) ^b^ | 1.015(1.004, 1.026) ^b^ |  | 0.992(0.987, 0.998) ^b^ | 0.985(0.974, 0.996) ^b^ | 1.008(1.002, 1.013) ^b^ | 1.015(1.004, 1.026) ^b^ |
| 1 | 0.996(0.993, 0.999) ^b^ | 0.991(0.985, 0.997) ^b^ | 1.004(1.001, 1.007) ^b^ | 1.009(1.003, 1.015) ^b^ |  | 0.988(0.980, 0.996) ^b^ | 0.976(0.960, 0.993) ^b^ | 1.012(1.004, 1.021) ^b^ | 1.024(1.007, 1.041) ^b^ |
| 2 | 0.998(0.996, 1.000) | 0.996(0.992, 1.001) | 1.002(1.000, 1.004) | 1.004(0.999, 1.008) |  | 0.986(0.977, 0.996) ^b^ | 0.973(0.954, 0.992) ^b^ | 1.014(1.004, 1.024) ^b^ | 1.028(1.008, 1.048) ^b^ |
| 3 | 1.000(0.998, 1.002) | 1.000(0.996, 1.005) | 1.000(0.998, 1.002) | 1.000(0.995, 1.004) |  | 0.986(0.976, 0.997) ^b^ | 0.973(0.953, 0.993) ^b^ | 1.014(1.003, 1.024) ^b^ | 1.028(1.007, 1.049) ^b^ |
| 4 | 1.001(0.999, 1.004) | 1.003(0.998, 1.007) | 0.999(0.996, 1.001) | 0.997(0.993, 1.002) |  | 0.988(0.977, 0.998) ^b^ | 0.976(0.955, 0.997) ^b^ | 1.012(1.002, 1.023) ^b^ | 1.025(1.003, 1.047) ^b^ |
| 5 | 1.002(1.000, 1.004) | 1.004(0.999, 1.008) | 0.998(0.996, 1.000) | 0.997(0.992, 1.001) |  | 0.989(0.978, 1.001) | 0.979(0.957, 1.001) | 1.011(0.999, 1.022) | 1.021(0.999, 1.045) |
| 6 | 1.002(0.999, 1.004) | 1.003(0.998, 1.008) | 0.998(0.996, 1.001) | 0.997(0.992, 1.002) |  | 0.991(0.979, 1.003) | 0.982(0.959, 1.005) | 1.009(0.997, 1.021) | 1.018(0.995, 1.042) |
| 7 | 1.001(0.996, 1.005) | 1.001(0.993, 1.010) | 0.999(0.995, 1.004) | 0.999(0.990, 1.007) |  | 0.992(0.979, 1.005) | 0.983(0.958, 1.009) | 1.008(0.995, 1.022) | 1.017(0.991, 1.044) |
| PM_2.5_ (μg/m^3^) |  |  |  |  |  |  |  |  |  |
| 0 | 0.996(0.993, 0.999) ^b^ | 0.992(0.985, 0.999) ^b^ | 1.004(1.001, 1.007) ^b^ | 1.008(1.001, 1.015) ^b^ |  | 0.996(0.993, 0.999) ^b^ | 0.992(0.985, 0.999) ^b^ | 1.004(1.001, 1.007) ^b^ | 1.008(1.001, 1.015) ^b^ |
| 1 | 0.996(0.993, 0.999) ^b^ | 0.992(0.986, 0.998) ^b^ | 1.004(1.001, 1.007) ^b^ | 1.008(1.002, 1.014) ^b^ |  | 0.992(0.986, 0.998) ^b^ | 0.984(0.972, 0.996) ^b^ | 1.008(1.002, 1.014) ^b^ | 1.016(1.004, 1.029) ^b^ |
| 2 | 0.996(0.994, 0.998) ^b^ | 0.992(0.988, 0.997) ^b^ | 1.004(1.002, 1.006) ^b^ | 1.008(1.004, 1.013) ^b^ |  | 0.988(0.980, 0.996) ^b^ | 0.976(0.960, 0.993) ^b^ | 1.012(1.004, 1.021) ^b^ | 1.024(1.007, 1.042) ^b^ |
| 3 | 0.996(0.994, 0.998) ^b^ | 0.992(0.988, 0.996) ^b^ | 1.004(1.002, 1.006) ^b^ | 1.008(1.004, 1.012) ^b^ |  | 0.984(0.974, 0.994) ^b^ | 0.968(0.949, 0.988) ^b^ | 1.016(1.006, 1.026) ^b^ | 1.033(1.012, 1.053) ^b^ |
| 4 | 0.996(0.994, 0.998) ^b^ | 0.992(0.989, 0.996) ^b^ | 1.004(1.002, 1.006) ^b^ | 1.008(1.004, 1.012) ^b^ |  | 0.980(0.969, 0.991) ^b^ | 0.961(0.939, 0.983) ^b^ | 1.020(1.009, 1.032) ^b^ | 1.041(1.018, 1.064) ^b^ |
| 5 | 0.996(0.994, 0.998) ^b^ | 0.992(0.988, 0.996) ^b^ | 1.004(1.002, 1.006) ^b^ | 1.008(1.004, 1.012) ^b^ |  | 0.976(0.964, 0.988) ^b^ | 0.953(0.930, 0.977) ^b^ | 1.024(1.012, 1.037) ^b^ | 1.049(1.024, 1.075) ^b^ |
| 6 | 0.996(0.994, 0.999) ^b^ | 0.992(0.987, 0.997) ^b^ | 1.004(1.001, 1.006) ^b^ | 1.008(1.003, 1.013) ^b^ |  | 0.973(0.960, 0.985) ^b^ | 0.946(0.921, 0.971) ^b^ | 1.028(1.015, 1.042) ^b^ | 1.057(1.030, 1.086) ^b^ |
| 7 | 0.996(0.993, 0.999) ^b^ | 0.992(0.986, 0.998) ^b^ | 1.004(1.001, 1.007) ^b^ | 1.008(1.002, 1.014) ^b^ |  | 0.969(0.955, 0.983) ^b^ | 0.938(0.912, 0.966) ^b^ | 1.032(1.017, 1.047) ^b^ | 1.066(1.035, 1.097) ^b^ |

^a^: The median concentration of SO_2_ is 9 μg/m^3^, so defined 0 μg/m^3^ as the concentration reduced by 10 μg/m^3^, there is no the concentration reduced by 20 μg/m^3^.

^b^: *P*<0.05.

**Table S8**  The single-day and cumulative effects of air pollutants on outpatients with psoriasis who elder than 65 years old when changed by 10 or 20 μg/m^3^.

| Lag (d) | Single-day effects | | | |  | Cumulative effects | | | |
| --- | --- | --- | --- | --- | --- | --- | --- | --- | --- |
|  | 10 units increment | 20 units increment | 10 units decrement | 20 units decrement |  | 10 units increment | 20 units increment | 10 units decrement | 20 units decrement |
| SO_2_ (μg/m^3^) ^a^ |  |  |  |  |  |  |  |  |  |
| 0 | 1.034(0.976, 1.096) | 0.980(0.868, 1.105) | 0.900(0.820, 0.988) ^b^ | - |  | 1.034(0.976, 1.096) | 0.980(0.868, 1.105) | 0.900(0.820, 0.988) ^b^ | - |
| 1 | 1.025(0.979, 1.073) | 0.978(0.889, 1.077) | 0.920(0.855, 0.991) ^b^ | - |  | 1.060(0.956, 1.176) | 0.959(0.772, 1.190) | 0.828(0.701, 0.978) ^b^ | - |
| 2 | 1.016(0.981, 1.052) | 0.977(0.907, 1.053) | 0.941(0.889, 0.995) ^b^ | - |  | 1.077(0.939, 1.235) | 0.937(0.702, 1.250) | 0.779(0.625, 0.972) ^b^ | - |
| 3 | 1.007(0.981, 1.034) | 0.976(0.920, 1.035) | 0.962(0.922, 1.004) | - |  | 1.085(0.924, 1.273) | 0.914(0.651, 1.283) | 0.750(0.579, 0.971) ^b^ | - |
| 4 | 0.998(0.975, 1.022) | 0.974(0.922, 1.029) | 0.983(0.946, 1.022) | - |  | 1.082(0.910, 1.288) | 0.890(0.613, 1.293) | 0.737(0.557, 0.977) ^b^ | - |
| 5 | 0.989(0.962, 1.017) | 0.973(0.912, 1.038) | 1.006(0.961, 1.052) | - |  | 1.071(0.894, 1.282) | 0.866(0.584, 1.286) | 0.741(0.553, 0.994) ^b^ | - |
| 6 | 0.980(0.945, 1.017) | 0.972(0.894, 1.056) | 1.028(0.968, 1.092) | - |  | 1.049(0.873, 1.261) | 0.842(0.556, 1.273) | 0.762(0.564, 1.029) | - |
| 7 | 0.971(0.926, 1.019) | 0.970(0.873, 1.079) | 1.051(0.972, 1.137) | - |  | 1.019(0.841, 1.235) | 0.817(0.524, 1.272) | 0.801(0.585, 1.097) | - |
| NO_2_ (μg/m^3^) |  |  |  |  |  |  |  |  |  |
| 0 | 1.036(1.018, 1.055) ^b^ | 1.067(1.035, 1.099) ^b^ | 0.958(0.934, 0.982) ^b^ | 0.913(0.862, 0.966) ^b^ |  | 1.036(1.018, 1.055) ^b^ | 1.067(1.035, 1.099) ^b^ | 0.958(0.934, 0.982) ^b^ | 0.913(0.862, 0.966) ^b^ |
| 1 | 1.028(1.014, 1.043) ^b^ | 1.053(1.028, 1.078) ^b^ | 0.968(0.948, 0.988) ^b^ | 0.934(0.892, 0.977) ^b^ |  | 1.066(1.033, 1.100) ^b^ | 1.123(1.065, 1.184) ^b^ | 0.927(0.886, 0.970) ^b^ | 0.852(0.769, 0.944) ^b^ |
| 2 | 1.020(1.009, 1.031) ^b^ | 1.039(1.020, 1.058) ^b^ | 0.978(0.963, 0.993) ^b^ | 0.955(0.922, 0.989) ^b^ |  | 1.088(1.043, 1.134) ^b^ | 1.167(1.087, 1.252) ^b^ | 0.906(0.853, 0.963) ^b^ | 0.814(0.710, 0.932) ^b^ |
| 3 | 1.012(1.004, 1.021) ^b^ | 1.025(1.012, 1.039) ^b^ | 0.988(0.976, 1.000) | 0.977(0.950, 1.004) |  | 1.101(1.048, 1.157) ^b^ | 1.196(1.101, 1.300) ^b^ | 0.896(0.834, 0.962) ^b^ | 0.795(0.677, 0.933) ^b^ |
| 4 | 1.005(0.997, 1.012) | 1.012(1.000, 1.024) ^b^ | 0.999(0.987, 1.010) | 0.999(0.974, 1.025) |  | 1.106(1.048, 1.167) ^b^ | 1.211(1.106, 1.325) ^b^ | 0.895(0.827, 0.967) ^b^ | 0.794(0.666, 0.947) ^b^ |
| 5 | 0.997(0.988, 1.005) | 0.999(0.985, 1.013) | 1.009(0.996, 1.023) | 1.022(0.992, 1.054) |  | 1.102(1.042, 1.166) ^b^ | 1.209(1.101, 1.328) ^b^ | 0.903(0.831, 0.980) ^b^ | 0.812(0.675, 0.977) ^b^ |
| 6 | 0.989(0.978, 1.000) ^b^ | 0.986(0.968, 1.004) | 1.020(1.002, 1.037) ^b^ | 1.046(1.005, 1.087) ^b^ |  | 1.090(1.029, 1.155) ^b^ | 1.192(1.084, 1.311) ^b^ | 0.920(0.845, 1.003) | 0.849(0.700, 1.030) |
| 7 | 0.981(0.967, 0.995) ^b^ | 0.973(0.950, 0.996) ^b^ | 1.030(1.008, 1.053) ^b^ | 1.070(1.017, 1.124) ^b^ |  | 1.070(1.007, 1.136) ^b^ | 1.160(1.051, 1.279) ^b^ | 0.948(0.866, 1.039) | 0.908(0.739, 1.116) |
| O_3_ (μg/m^3^) |  |  |  |  |  |  |  |  |  |
| 0 | 0.997(0.990, 1.004) | 0.994(0.980, 1.008) | 1.003(0.996, 1.010) | 1.006(0.992, 1.021) |  | 0.997(0.990, 1.004) | 0.994(0.980, 1.008) | 1.003(0.996, 1.010) | 1.006(0.992, 1.021) |
| 1 | 0.998(0.993, 1.004) | 0.996(0.985, 1.008) | 1.002(0.996, 1.007) | 1.004(0.992, 1.015) |  | 0.995(0.983, 1.008) | 0.990(0.965, 1.016) | 1.005(0.992, 1.018) | 1.010(0.984, 1.036) |
| 2 | 0.999(0.995, 1.004) | 0.999(0.991, 1.007) | 1.001(0.996, 1.005) | 1.001(0.993, 1.010) |  | 0.995(0.978, 1.012) | 0.989(0.956, 1.023) | 1.005(0.989, 1.023) | 1.011(0.977, 1.046) |
| 3 | 1.001(0.998, 1.004) | 1.001(0.995, 1.008) | 0.999(0.996, 1.002) | 0.999(0.992, 1.005) |  | 0.995(0.976, 1.015) | 0.991(0.953, 1.031) | 1.005(0.985, 1.025) | 1.009(0.970, 1.050) |
| 4 | 1.002(0.999, 1.005) | 1.004(0.998, 1.010) | 0.998(0.995, 1.001) | 0.996(0.990, 1.002) |  | 0.997(0.976, 1.019) | 0.995(0.953, 1.038) | 1.003(0.982, 1.024) | 1.005(0.963, 1.049) |
| 5 | 1.003(1.000, 1.007) | 1.007(1.000, 1.014) | 0.997(0.993, 1.000) | 0.993(0.987, 1.000) |  | 1.001(0.979, 1.023) | 1.001(0.958, 1.046) | 0.999(0.978, 1.022) | 0.999(0.956, 1.044) |
| 6 | 1.005(1.000, 1.009) | 1.009(1.000, 1.019) | 0.995(0.991, 1.000) | 0.991(0.982, 1.000) |  | 1.005(0.983, 1.028) | 1.011(0.966, 1.057) | 0.995(0.973, 1.017) | 0.990(0.946, 1.035) |
| 7 | 1.006(1.000, 1.012) | 1.012(1.000, 1.024) | 0.994(0.988, 1.000) | 0.988(0.977, 1.000) |  | 1.011(0.988, 1.035) | 1.022(0.976, 1.071) | 0.989(0.966, 1.012) | 0.978(0.934, 1.025) |
| PM_2.5_ (μg/m^3^) |  |  |  |  |  |  |  |  |  |
| 0 | 0.997(0.990, 1.004) | 0.994(0.980, 1.008) | 1.003(0.996, 1.010) | 1.006(0.992, 1.020) |  | 0.997(0.990, 1.004) | 0.994(0.980, 1.008) | 1.003(0.996, 1.010) | 1.006(0.992, 1.020) |
| 1 | 0.998(0.992, 1.003) | 0.995(0.984, 1.007) | 1.002(0.997, 1.008) | 1.005(0.993, 1.016) |  | 0.995(0.982, 1.008) | 0.990(0.965, 1.015) | 1.005(0.992, 1.018) | 1.010(0.985, 1.036) |
| 2 | 0.998(0.994, 1.003) | 0.997(0.988, 1.006) | 1.002(0.997, 1.006) | 1.003(0.994, 1.012) |  | 0.993(0.976, 1.010) | 0.987(0.954, 1.021) | 1.007(0.990, 1.024) | 1.014(0.980, 1.049) |
| 3 | 0.999(0.995, 1.003) | 0.998(0.991, 1.005) | 1.001(0.997, 1.005) | 1.002(0.995, 1.009) |  | 0.992(0.972, 1.012) | 0.984(0.946, 1.025) | 1.008(0.988, 1.028) | 1.016(0.976, 1.058) |
| 4 | 1.000(0.996, 1.003) | 0.999(0.992, 1.006) | 1.000(0.997, 1.004) | 1.001(0.994, 1.008) |  | 0.992(0.970, 1.014) | 0.983(0.940, 1.028) | 1.008(0.986, 1.031) | 1.017(0.972, 1.063) |
| 5 | 1.000(0.996, 1.004) | 1.000(0.992, 1.008) | 1.000(0.996, 1.004) | 1.000(0.992, 1.008) |  | 0.992(0.968, 1.016) | 0.984(0.938, 1.032) | 1.008(0.984, 1.033) | 1.017(0.969, 1.066) |
| 6 | 1.001(0.996, 1.006) | 1.001(0.991, 1.012) | 0.999(0.994, 1.004) | 0.999(0.989, 1.009) |  | 0.993(0.968, 1.018) | 0.985(0.936, 1.037) | 1.008(0.982, 1.034) | 1.015(0.965, 1.068) |
| 7 | 1.001(0.995, 1.008) | 1.003(0.990, 1.015) | 0.999(0.992, 1.005) | 0.997(0.985, 1.010) |  | 0.994(0.967, 1.022) | 0.988(0.935, 1.044) | 1.006(0.979, 1.034) | 1.012(0.958, 1.070) |

^a^: The median concentration of SO_2_ is 9 μg/m^3^, so defined 0 μg/m^3^ as the concentration reduced by 10 μg/m^3^, there is no the concentration reduced by 20 μg/m^3^.

^b^: *P*<0.05.
